# Noninvasive, label-free image approaches to predict multimodal molecular markers in pluripotency assessment

**DOI:** 10.1038/s41598-024-66591-z

**Published:** 2024-07-09

**Authors:** Ryutaro Akiyoshi, Takeshi Hase, Mayuri Sathiyananthavel, Samik Ghosh, Hiroaki Kitano, Ayako Yachie

**Affiliations:** 1 Yokogawa Electric Corporation, 2-9-32 Nakacho, Musashino-shi, Tokyo, 180-8750 Japan; 2 The Systems Biology Institute, Saisei Ikedayama Bldg., 5-10-25, Higashi Gotanda, Shinagawa-ku, Tokyo, 141-0022 Japan; 3 SBX BioSciences, Inc, 1111 West Georgia Street, 20th Floor, Vancouver, BC V6E 4G2 Canada

**Keywords:** Regenerative medicine, Induced pluripotent stem cells, Image processing, Machine learning

## Abstract

Manufacturing regenerative medicine requires continuous monitoring of pluripotent cell culture and quality assessment while eliminating cell destruction and contaminants. In this study, we employed a novel method to monitor the pluripotency of stem cells through image analysis, avoiding the traditionally used invasive procedures. This approach employs machine learning algorithms to analyze stem cell images to predict the expression of pluripotency markers, such as OCT4 and NANOG, without physically interacting with or harming cells. We cultured induced pluripotent stem cells under various conditions to induce different pluripotent states and imaged the cells using bright-field microscopy. Pluripotency states of induced pluripotent stem cells were assessed using invasive methods, including qPCR, immunostaining, flow cytometry, and RNA sequencing. Unsupervised and semi-supervised learning models were applied to evaluate the results and accurately predict the pluripotency of the cells using only image analysis. Our approach directly links images to invasive assessment results, making the analysis of cell labeling and annotation of cells in images by experts dispensable. This core achievement not only contributes for safer and more reliable stem cell research but also opens new avenues for real-time monitoring and quality control in regenerative medicine manufacturing. Our research fills an important gap in the field by providing a viable, noninvasive alternative to traditional invasive methods for assessing pluripotency. This innovation is expected to make a significant contribution to improving regenerative medicine manufacturing because it will enable a more detailed and feasible understanding of cellular status during the manufacturing process.

## Introduction

Pluripotent stem cells (PSCs), including embryonic stem cells (ESCs) and induced pluripotent stem cells (iPSCs), produce all cell types derived from the three germ layers. Given their indefinite proliferative and differentiation potential, PSCs are gaining attention in various fields including regenerative medicine, drug testing, disease modeling, and embryonic development. Detecting PSCs that escape pluripotency maintenance or complete differentiation to specific lineages is critical to ensure their quality in subsequent studies^1^.

Instead of direct pluripotency assessment by introducing PSCs into a developing embryo and subsequent assessment of chimerism or checking for the viability of PSCs-derived organisms^2^, several marker genes or proteins are commonly used to identify and characterize PSCs. Octamer-binding transcription factor 4 (**OCT4)**, also known as POU domain, class 5, transcription factor 1 (POU5F1), is one of the earliest markers of pluripotent cells, and is essential for maintaining self-renewal and pluripotency by repressing genes associated with differentiation and promoting the expression of pluripotency-associated genes. Another key transcription factor, **NANOG**, cooperates with other interconnected factors to establish naive and undifferentiated state of pluripotent cells^3–5^. Although both OCT4 and NANOG are involved in regulating pluripotency, as well as each other, their differential-expression and function contribute to the complexity and robustness of pluripotency regulation in embryonic cells and iPSCs^6^. In addition to these intracellular regulators of pluripotency, several cell surface markers expressed on undifferentiated pluripotent cells and downregulated upon differentiation, such as stage-specific embryonic antigen 4 (**SSEA-4**) and tumor-related antigen 1–60 (Tra-1-60), play key roles in the identification and isolation of PSCs during flow-cytometric cell sorting^7^.

However, traditional assessment methods for molecular markers often involve invasive techniques, such as Flow Cytometry (FCM), quantitative Polymerase Chain Reaction (qPCR), RNA sequencing (RNA-Seq), and immunostaining, and may limit their use in quality control evaluation when direct clinical application of these cells is intended. While the number of cells lost in a single test may not be significant, the cumulative loss of cells becomes considerable when these invasive tests are repeatedly conducted over a long-term cell manufacturing process. These challenges become particularly critical in scenarios where the manufacturing process demands consistent and long-term testing, thereby compounding the challenge of sustainable and efficient production and highlighting the need for noninvasive approaches^8–10^. In this study, we focused on image analysis using bright-field microscopy, which is routinely employed to observe cell morphology and detect variations in cellular states. Bright-field microscopy is noteworthy for noninvasive analysis because it is cost-effective and does not require analytical reagents. Recent studies have explored the potential of bright-field microscopy to assess stem cell quality. Notably, differences were observed in the morphology, motility, and proliferation rate of iPSC progenitors and mouse embryonic fibroblasts. These differences enable early identification of iPSC progenitors during reprogramming using machine learning techniques^11^. Despite the noninvasive advantage of assessing cell quality based on morphology, reliance on previously identified morphological features for discriminating cellular states presents limitations, particularly in the differentiation of iPSCs and their application in internal organ therapies.

Christiansen et al. proposed a model that predicts CD31 protein expression from phase-contrast images by acquiring CD31 protein-stained fluorescence images of iPSCs and derived endothelial cells^12^. This model facilitates the distinction between iPSCs and differentiated endothelial cells based on predicted CD31 protein expression levels. However, this approach requires labeling of immune-stained fluorescence images and corresponding cell annotation by experts.

To address these limitations, Schmauch et al. reported a method for predicting cancer-related gene expression levels in tissue images^13^. This method correlates pathological section images of cancer with RNA-Seq data, circumventing the need for cell morphology identification and individual cell annotation. Although high accuracy in predicting RNA-Seq data from bright-field images of tissues has been achieved, this method relies on stained and fixed cell images and is thus invasive.

Therefore, we developed a model to predict the expression of molecular pluripotency markers from live cell images obtained using bright-field microscopy without staining and prior expert annotation. This method involves building classification models from bright-field cell images using unsupervised or semi-supervised modeling frameworks. Using the resulting models with different parameters and preprocessing methods, we identified a model that could accurately predict the outcomes of several molecular assays.

## Materials and methods

### iPSC cultures

iPSCs (201B7, 1231A3 strains, from the Center for iPS Cell Research and Application, Kyoto University) were maintained in StemFit (Ajinomoto Co., Inc., Tokyo, Japan) medium with 10 μM Y-27632 (FUJIFILM Wako Pure Chemical Corporation, Osaka, Japan) on culture dishes coated with iMatrix511 (Nippi, Incorporated, Tokyo, Japan). iPSCs (ND50018, ND50019 strains, from the NINDS Human Cell and Data Repository, NIH) were maintained in mTeSR Plus (Stem Cell Technologies, Vancouver, Canada) medium on Matrigel Matrix Basement Membrane Growth Factor Reduced (Corning, New York, USA) coated culture dishes. The next day, on Days 3, 5, and 6, the medium was replaced with 1.5 mL StemFit or mTeSR Plus, and iPSCs were passaged within 7 days. iPSCs were passaged by washing in PBS, incubated in Accutase (Stem Cell Technologies, Vancouver, Canada) for 3 min at 37 °C, detached by pipetting, harvested, and reseeded to a count of 1.0 × 10^5^ cells/well in StemFit or mTeSR Plus with 10 μM Y-27632 on culture dishes coated with iMatrix511 or Matrigel. Four culture conditions were used for following iPSC maintenance and propagation: (1) Control: cultured using the general iPSC maintenance culture method; (2) low nutrient: cultured in inactivated medium (StemFit medium with heat treatment at 56 °C for 30 min); (3) Fetal Bovine Serum (FBS) condition: cultured using differentiation medium (DMEM supplemented with 10% FBS, 1% MEM NEAA, and 1% GlutaMax); and (4) physical stimulus: suspended by pipetting 20 times at passaging and cultured using the general iPSC maintenance culture method. The examination of the four conditions was conducted using the 201B7 strain. Other cell strains were cultured only under Condition 1. Microscopic imaging was performed on days 1 and 4 under the four conditions, and various evaluations (qPCR, flow cytometry, immunocytochemistry, and RNA-Seq) were performed on the cells after imaging on Day 5. Each condition was allocated to a single well within a 6-well plate, with this configuration replicated across five identical plates to support comprehensive analysis through various methodologies. The distribution of conditions and analytical techniques employed is detailed below:

Bright-field Imaging: One plate was reserved for bright-field microscopy to observe and document cellular morphology. Immunostaining: Two plates were subjected to immunostaining, aimed at detecting specific proteins and assessing their localization within cells. Molecular Analyses: The remaining two plates were used for advanced molecular analyses. qPCR was performed to quantify gene expression levels. FCM was utilized to analyze protein expression. RNA-Seq provided comprehensive gene expression profiles. This experimental arrangement was executed in triplicate (N = 3) to ensure statistical robustness and reproducibility of the findings. Each iteration of the experiment was prepared and analyzed under identical conditions, allowing for direct comparison across trials.

### Cell viability assessment

We measured the viability of cells harvested on Day 5 using a ViCELL XR Cell Viability Analyzer (Beckman Coulter, Inc.) in accordance with the manufacturer’s standard protocol. The trypan blue exclusion method was used with ViCELL XR for automated analysis of cell viability. The cells, prepared at a concentration of 1 × 10^6^ cells/mL and mixed with trypan blue, were analyzed to determine the percentage of viable and nonviable cells.

### Imaging

Images of cells cultured in 6-well plates (Corning Incorporated, Corning, USA) were acquired with 20 × high numerical aperture objectives using CQ1 (Yokogawa Electric Corporation). In four wells, five 5 × 8 square regions were placed in 200 fields (field of view (FOV)) for image acquisition. In each field, 5 planes with a distance of 5 μm between each plane in the Z-axis were acquired. Bright-field images (50-ms exposure) were acquired over time-lapse, with an interval of 1 h and total acquisition time of 20 h. Multiple bright-field images captured by moving the objective lens along the axis normal to the cell plane were substituted into the light-propagation equation. The refractive index differs between cells and the background, which leads to differences in the optical phases. Based on this fact, cellular morphology is calculated using the light-propagation equation^14^ to create contrast-enhanced (CE) bright-field images. All bright-field images were converted to CE bright-field images using the High Content Analysis Software System CellVoyager CellPathfinder (Yokogawa Electric Corporation). This conversion is in sharp contrast to the image of clear cells that enables their observation.

### Flow cytometry

The cells were dissociated into single cells using Accutase (Stem cell technology), suspended in PBS, and stained with a 1:1000 dilution of Fixable Viability Stain 660 (Becton Dickinson, Franklin Lakes, USA), according to the manufacturer’s instructions. The cells were collected by centrifugation, washed with PBS, fixed with 4% PFA, washed with PBS, and collected by centrifugation. The cells were then stained with FITC mouse anti-human TRA-1-60 antibody (Becton Dickinson, #560380) diluted 1:20 and Alexa Fluor 555 mouse antibody SSEA-4 (Becton Dickinson, #560218) diluted 1:10 in FACS Buffer. Live cells were analyzed using BD FACS Aria Fusion (Becton Dickinson).

### Flow cytometry data analysis

To quantify TRA-1-60 and SSEA-4 double-positive cells, FCM data analysis was performed in two ways: manual- and auto-gating. The auto-gating approach used the OpenCyto package (version 2.2) in R (version 4.0.3) with Bioconductor software (version 3.12). First, a singlet gate function was applied to the root population to remove doublets with default setting using FSC-A and FSC-H. In the singlet population, the tailgate function was applied to remove debris and to include live cells with a tolerance of 1e-8 for the tailgate. Then the function of mindensity2 was applied to gate the windows and obtain the largest cell population with the staining channels TRA-1-60 and SSEA-4, where gating windows 1–8 for log(e) values were used for mindensity2. The gate-range parameter in mindensity2 was set by using unstained control as a reference for automatic identification of the background florescence range. The results of the FCM analysis were visualized using the ggcyto package (version 1.18) in R. Manual-gating analysis was performed using the BD FACSAria Fusion system (version 1.0, BD Biosciences). First, after Area-scaling, single cells were selected using the forward-scatter area (FSC-A) and side-scatter area (SSC-A) to exclude debris. FSC-A determines cell size and SSC-A assesses internal complexity by removing smaller, less complex debris. Second, viable cells were separated using cells stained with Fixable Viability Stain 660, which penetrates the cell membrane and marks nonviable cells with detectable fluorescence in the APC-A channel, distinguishing them from viable cells. Non-stained controls were used to set thresholds and ensure accurate gating. Live cells were separated using a manual-gating approach with a static polygon-type filter through the LiveCell filter in the software. Subsequently, TRA-1-60 and SSEA-4 filters were used to identify cells expressing pluripotency markers and analyze specific subpopulations with the gates by manually drawing closed polygon shapes on the plot. The antibodies used in the FCM are listed in Supplementary Table 1.

### Immunochemistry and quantification

Cells were fixed in 4% paraformaldehyde (FUJIFILM Wako Pure Chemical Corporation) for 15 min at room temperature, washed with PBS, permeabilized in 0.2% TritonX-100/PBS (Sigma-Aldrich, St. Louis, USA) for 15 min, and blocked with 1% BSA for 30 min. The cells were probed at 4 °C overnight with Oct 3/4 Antibody (Santa Cruz Biotechnology, #sc5279) diluted 1:100 and anti-NANOG antibody, clone 7F7.1 (Sigma-Aldrich, #MABD24) diluted 1:500. The cells were then washed thrice in PBS, labeled for 1 h with donkey anti-mouse IgG (H + L) Alexa Fluor 488 (Thermo Fisher Scientific, #A21202) diluted 1:1000 and Hoechst 33,342 (Thermo Fisher Scientific, #H3570) diluted 1:1000, and imaged using a Confocal Quantitative Image Cytometer CellVoyager CQ1 (Yokogawa Electric Corporation, Tokyo, Japan). Information on the primary and secondary antibodies is provided in Supplementary Table 2. The fluorescence intensity in each cell was quantified using CellPathfinder software ver.R3.06.02 (Yokogawa Electric Corporation). Initially, dual-stained samples with Hoechst 33,342 and Oct3/4 antibody were processed. Nuclei in these samples were identified according to an analytical protocol for nucleus detection, employing data from Hoechst 33342 dye observed through Channel 1, characterized by an excitation wavelength of 405 nm and an emission bandpass filter of 444/60 nm. Following nuclear identification in Channel 1, the regions of interest were delineated within the nuclear recognition fields. Within these regions of interest, the fluorescence intensity was measured for Channel 2 (excitation: 488 nm, emission: bandpass 525/50 nm) to quantify Oct3/4 expression in individual cells. A comparable methodology was subsequently applied to the Hoechst 33342 and anti-NANOG antibody dual-stained samples. In these samples, NANOG expression levels were quantified using the same fluorescence parameters as in Channel 2.

### Immunochemistry data analysis

To quantify the ratio of marker protein-positive cells based on immunochemistry intensity data, manual and automated threshold binarization methods were applied. Arbitrary manual thresholds of 100 and 80 were adopted for the intensities of NANOG and OCT4, respectively, to separate the two peaks of bimodal intensity histograms based on the profiles in Cond 1, which filtered out cells with no to very low intensity. As an automated method, the Binarization Across Multiple Scales (BASC) algorithm was employed to determine the threshold for each dataset^15^, which is implemented in binarize. BASC function in the R library binarize version 1.3.1. Briefly, BASC-based binarization was performed using the following steps: (1) sort the intensity values in order from smallest to largest, (2) approximate intensity values with a step function tuned to have as few discontinuities as possible and represent the original values as much as possible, and (3) find the point of strongest discontinuity based on a step function in sorted intensities.

### Real-time quantitative PCR

RNA was prepared using an RNeasy Mini Kit (QIAGEN N.V., Venlo, Nederland) according to the manufacturer’s instructions. RNA was reverse-transcribed using ReverTra Ace qPCR RT Master Mix Reagents (Toyobo Co., Ltd., Osaka, Japan). Real-time RT-PCR was performed using the TaqMan Fast Advanced Master Mix (Thermo Fisher Scientific) and QuandStudio5 real-time PCR device (Thermo Fisher Scientific). PCR data were normalized to *ActB* expression and the comparative Ct quantitation method was used. The ΔCt value of cells cultured under condition 1 was used as the reference sample for the calculation of 2^−ΔΔCt^. Primers, probes, and thermal-cycling conditions are listed in Supplementary Table 3.

### RNA-Seq analysis

Total RNA was extracted from iPSCs using the RNeasy Mini Kit (Qiagen) following the manufacturer’s instructions. The libraries were prepared using a NEBNext Poly(A) mRNA Magnetic Isolation Module (#E7490) and a NEBNext Ultra II Directional RNA Library Prep Kit (#E7760) and sequenced using Illumina NovaSeq 6000 (Illumina, USA). Fastq files were generated using the bcl2fastq tool (v. 2.20.0.422). For quality control, polyA/T sequences, adaptor sequences, and poor quality bases were removed using cutadapt (4.1)^16^ and fastp (v. 0.20.1)^17^. The trimmed reads were aligned to the human genome assembly GRCh38 using the HiSAT2 tool (v. 2.1.0)^18^. Mapped reads on the annotated genes were counted using featureCounts^19^. Read count was converted to transcripts per million for subsequent analysis. The RNA-Seq dataset is publicly available at GEO with accession number GSE256303.

### RNA-Seq data analysis to identify differences between experimental conditions

Differentially expressed genes (DEGs) were obtained from RNA-Seq analysis using the limma package v3.54.2 on R version 4.2.2, where Cond 2, Cond 3, and Cond 4 samples were compared with the control samples (Cond 1). Genes with absolute fold change (FC) > 2 and *p*-value < 0.05 were taken as DEGs for subsequent analysis. The results of the DEG analysis were displayed using a volcano plot using the R package ggplot2 v3.4.2. Principal component analysis (PCA) was performed using scikit-lean v1.2.2 in.

Python to show the variance between experimental groups. Gene set enrichment analysis (GSEA) of each experimental group compared to the control group was performed using the R package clusterProfiler v4.6.2^20^ to identify biological processes (Gene Ontology 3.14.0, downloaded in September 2023).

### Statistical test and correlation analysis

Welch’s *t*-test was used to determine the significance of difference in mean values between Cond 1 and the other groups with t.test function in R version 4.2.0. Correlation analysis was performed using the pandas package version 1.5.3 in python.

### Image preprocessing

Images were resized using the function of the python module Pillow (version 6.2.1). For brightness tuning, the mean luminance of the images was aligned to 128 and the standard deviation was 32, *i*.*e*., the value of the brightness of each pixel in each input image was tuned using the following equation:

$${nb}_{j,i}=\left(\frac{{b}_{j,i}-{\mu }_{i}}{{\sigma }_{i}}\right)*32-128,$$

Where ${nb}_{j,i}$, ${b}_{j,i}$, ${\mu }_{i}$, and ${\sigma }_{i}$ are the values of tuned brightness of pixel *j* in image *i*, the value of brightness of pixel *j* in image *i*, mean value of brightness among all pixels in image *i*, and standard deviation value of brightness among all pixels in image *i*, respectively. The mean and standard deviation were calculated using Numpy version 1.21.4. Cell cropping was performed using the pretrained U-Net models in the HPA Cell Segmentation module^21^ to segment FOV images. The resultant single-cell images of > 1000 pixels and < 100,000 pixels were used for classification.

### Image classifier framework

In the image classifier framework using a one-class support vector, dimensionality reduction was performed using uniform manifold approximation and projection (UMAP) with umap-learn version 0.5.1 onto preprocessed tile or cell images to embed the images into three-dimensional features. OneClassSVM, a function of scikit-learn version 0.24.1 in python version 3.8.8, with default parameter values, except nu, was applied to the UMAP dimensions. For other existing CNN model-based frameworks, we rebuilt the ResNet50 model using the training dataset of a study^22^. The semi-supervised learning pipeline was built using pytorch version 1.9.0, fastai version 1.0.60 in python version 3.6.9.

### Model robustness analysis

Robustness analysis of the well-level model pluripotency prediction against the reduction in the number of input FOVs was performed by comparing the calculated pluripotency ratios between using all FOVs (400 FOVs) and randomly selecting 10% of the total FOVs (40 FOVs) of the well-image, which were repeated 100 times.

## Results

### Distinct conditions to culture iPSCs

**Figure 1:**
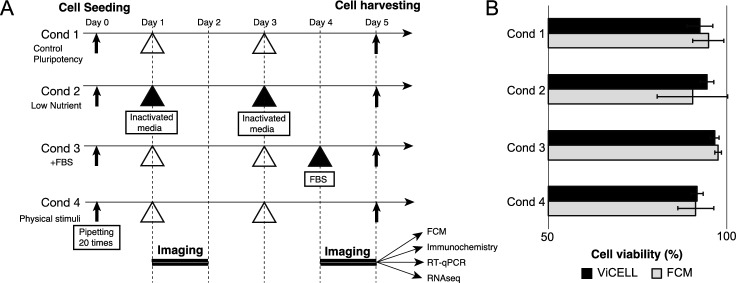
Experimental arrangement of distinct culture conditions. (**A**) For all four conditions, cell imaging was performed on Days 1–2 and Days 4–5 after cell seeding. White and black triangles indicate cell media change to pluripotency media and condition-specific media, respectively. Cells were harvested on Day 5, followed by molecular assessment. (**B**) The viability of cells harvested on Day 5 was measured using ViCELL XR Cell Viability Analyzer and FCM.

To observe variations in pluripotency status of iPSCs under distinct culture conditions and different molecular measurements, we defined an experimental arrangement (Fig. 1A). Cell viability analysis showed a nonsignificant decrease in cell viability in Cond 2 and Cond 4 cells (Fig. 1B**)**. Microscopic imaging was performed at 20 time points on days 1 and 4 after cell seeding. Cells from all replicates in each condition were harvested on Day 5, distributed, and used for molecular assay-based pluripotency assessment using flow cytometry, immunocytochemistry, qPCR, and RNA-Seq. Three technical replicates were tested for each condition.

### Image data acquisition of iPSCs

**Figure 2:**
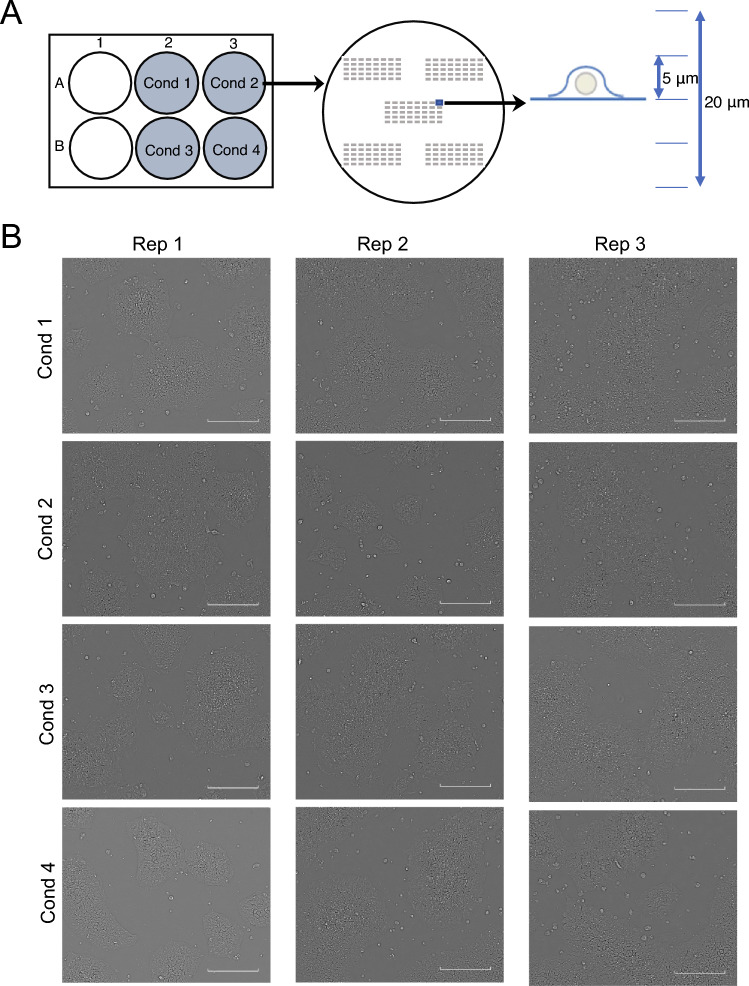
Image data acquisition pipeline. (**A**) Plate arrangement for four conditions (left). There are five 5 × 8 square regions for 200 fields of view (FOVs) in a single well (middle). In each field, five planes were acquired at 5-μm intervals along the Z-axis, which were overlaid into a single image for use in image analysis (right). (**B**) Representative FOVs of the replicates in each of the four conditions in the same coordinate. We selected the image from the 88th FOV. A scale bar of 200 μm is shown at the bottom right. The original images were available as Supplementary File.

For cell imaging, we employed time-lapse bright-field imaging and created the final cell image (Fig. 2A) using a commercial software called CellPathfinder. The resulting image dataset for each sample comprised 400 FOVs. Representative FOV images of each sample are shown in Fig. 2B. The morphological differences in iPSCs among Cond 1, Cond 2, and Cond 4 were undetected through visual inspection by human experts in iPSC maintenance. In contrast, in Cond 3, morphological transformation of the cells was visible, where the outer contour of each cell was clearer, the cell shape was flattened, and confluence was higher.

### Multimodal molecular data acquisition of iPSCs

**Figure 3:**
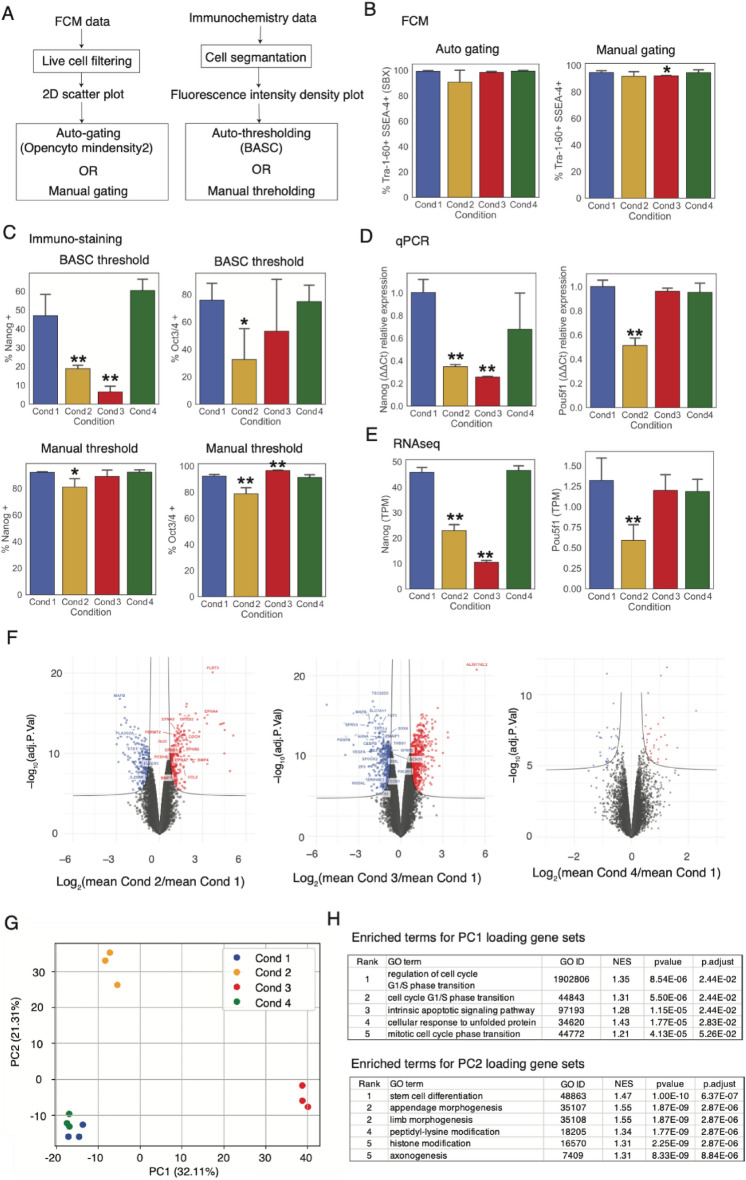
Pluripotency assessment by molecular measurements using different molecular assays. (**A**) Workflows of FCM and immunochemistry data processing. Each sample was assessed using manually and computationally determined values to split the data points into pluripotency or nonpluripotency. (**B**) Pluripotency ratio assessed in SSEA-4 and Tra-1–60 double-positive cells by FCM analysis. (**C**) Pluripotency ratio assessed with NANOG- and OCT4-positive cells by immunochemistry. (**D**,**E**) Gene expression levels of *NANOG* and *POU5F1* assessed using qPCR and RNA-Seq. (**F**) Volcano plots of RNA-Seq data (TPM) between Cond 1 and other conditions, where X- and Y-axes indicate Log2-transformed fold change and Log10-transformed adjusted *p*-values, respectively. Genes with expression (TPM) < 1 in any of the 12 samples were excluded before plotting. (**G**) PCA plot of RNA-Seq data, where TPM value was used as input. (**H**) Top five enriched pathways for PC1 and PC2 loading genes analyzed by GSEA against GO biological processes gene sets. In panels (**B**–**E**) single asterisks indicate *p*-value < 0.1, and double asterisks indicate *p*-value < 0.05 by Welch’s *t*-test, comparing the means of three replicates of control (Cond 1) and other conditions, respectively.

In FCM analysis, we employed SSEA-4 and Tra-1-60 as cell surface markers for pluripotency enrichment. The FCM-derived pluripotency ratio was defined as the percentage of double-positive cells per total live cells based on manual- or auto-gating methods (described in the Methods section) (Fig. 3A, Supplementary Fig. 1). As a result, the pluripotency ratio remained relatively high with both manual- and auto-gating, and the mean values of all conditions were > 90% (Fig. 3B). The mean pluripotency ratio of the three replicates in Cond 2 was lower than that in the control condition with manual- and auto-gating, but was not significant due to large intra-condition variance. With manual-gating, the decrease in the pluripotency ratio in Cond 3 cells was slightly significant (*p* < 0.1, Welch’s *t*-test), whereas the decrease in the ratio in Cond 3 cells was not observed with auto-gating. The other automated gating function, tailgate, which is suitable for detecting minimum cutpoint in only one major peak, was tested whether the results were common with mindensity2-based automated gating (Supplementary Fig. 2). Although the decrease in positive cells in Cond2 and Cond4 were emphasized in the tailgate-based pipeline, the positive cell profiles in two automated pipelines (mindensity2 and tailgate) were well correlated (*r* = 0.95), and the impacts of the downstream analysis in comparison with model prediction are supposed to be minimal.

Immunostaining was performed for proteins immunoprecipitated using antibodies against OCT4 and NANOG. Based on the density plot of the fluorescence intensity of each protein per cell, manual and automated thresholding was applied to eliminate arbitrariness (Fig. 3A, Supplementary Fig. 3). To use an automated binarization approach, we employed the BASC algorithm. The number of positive cells with an intensity above each threshold per total cell number was calculated for each sample as an immunochemistry-derived pluripotency ratio indicated by each marker protein (Fig. 3C). The NANOG-positive cell ratio in Cond 1 cells with the BASC threshold was lower than that with the manual threshold. At the BASC threshold, Cond 2 and Cond 3 showed significantly decreased positive cell ratios. The depletion in the ratio was clearer in Cond 3. In contrast, with the manual threshold, the decrease was moderately significant in Cond 2 only (adjusted *p*-value < 0.1) and the mean profile was similar to that in FCM-assessed pluripotency with manual-gating (Pearson’s correlation coefficient was 0.93). The OCT4-positive cell ratio in Cond 1 with the BASC threshold was lower than that with the manual threshold, whereas the overall positive cell ratio was higher in OCT4 than that in NANOG. Consistently, NANOG exhibited a high degree of heterogeneity with a broad distribution of expression values in maintained pluripotent cells, whereas OCT4 exhibited more uniform expression^23^. The BASC threshold resulted in slightly significant and nonsignificant decreases in the mean positive cell ratios for Cond 2 and Cond 3, respectively. In contrast, the manual threshold showed significant decrease and significant increase in OCT4-positive cell ratios in Cond 2 and Cond 3, respectively.

To further assess the differences in intracellular pluripotency markers across conditions, we performed qPCR for *POU5F1* and *NANOG*, as well as RNA-Seq. In qPCR analysis, Cond 2 showed significant depletion of *NANOG* and *POU5F1* expression compared to the control conditions (left and right panels Fig. 3D, respectively). In contrast, the decrease in *NANOG* expression was more intense in Cond 3 than in Cond 2, and *POU5F1* expression in Cond 3 was unchanged compared that to in Cond 1. Consistent results were obtained with the RNA-Seq analysis of *NANOG* and *POU5F1* expression (Fig. 3E). Moreover, the *NANOG* expression pattern was highly correlated with the FCM-based pluripotency ratio with manual-gating and immunostaining-derived NANOG-positive cell ratio with BASC thresholding. The *POU5F1* expression pattern was correlated with the FCM-based pluripotency ratio with auto-gating and immunostaining-derived NANOG-positive cell ratio with manual thresholding. Interestingly, a nonsignificant but considerable decrease and large variation among samples was observed in *NANOG* expression by qPCR. The results of the molecular assessment of pluripotency markers are listed in Supplementary Table 4.

The differential-expression volcano plots of RNA-Seq data indicated that many genes were significantly up- or downregulated in Cond 2 and Cond 3 and changed little in Cond 4 compared with Cond 1 (Fig. 3F). Interestingly, many pathways related to cell morphology, cell number, cytoskeleton, cell size, and cell adhesion were enriched as significantly up- and downregulated pathways in Cond 2 and Cond 3, respectively (Supplementary Table 5). The genes involved in these pathways are mapped to a volcano plot (Fig. 3F).

PCA results of RNA-Seq data concurred with the difference in the cellular states cultured in Cond 2 and Cond 3, indicating a possible deviation of cell populations from the pluripotent state (Fig. 3G). Changes in cell status in Cond 2 and Cond 3 due to pluripotency are described by variations along different PC axes, PC2 and PC1, respectively. The GSEA results of the PC1 gene set indicated that culture in differentiation media in Cond 3 led to changes in cell cycle-related biological processes, and lowered nutrients in Cond 2 stimulated the gene set to be highly enriched in stem cell differentiation (adjusted *p*-value is low: 2.87e-6) and appendage and limb morphogenesis, as indicated in the GSEA results of the PC2 gene set (Fig. 3H).

Thus, Cond 2 is characterized by a consistently low expression of Nanog and Oct4 genes and proteins at single-cell and population levels, which may have been caused or influenced by changes in the expression of genes related to stem cell differentiation. However, Cond 3 decreased Nanog protein and gene expression but did not significantly change Oct4 protein and gene expression at either the individual cell or population levels. The depletion of Nanog in Cond 3 was significant even in comparison with Cond 2 in BASC-based protein quantification by immunostaining (*p*-value = 0.007 and adjusted *p*-value = 0.028 for Cond 2 vs. Cond 3), gene expression in qPCR (*p*-value = 0.006, adjusted *p*-value = 0.012), and RNA-Seq (*p*-value = 0.007, adjusted *p*-value = 0.010). In contrast, Oct4 expression was higher in Cond 3 than in Cond 2 in the manual thresholding of immunostaining (*p*-value = 0.021, adjusted *p*-value = 0.039 for Cond 2 vs. Cond 3), qPCR (*p*-value = 0.002, adjusted *p*-value = 0.005), and RNA-Seq (*p*-value = 0.017, adjusted *p*-value = 0.048).

Contrary to the clear depletion in the intracellular molecular markers in Cond 2, the decrease in the FCM-based pluripotency ratio based on cell surface markers was not significant under these conditions, although there was a decreasing trend. Instead, FCM analysis with manual thresholding showed a decreased pluripotency ratio in Cond 3.

### Image AI model-building

**Figure 4:**
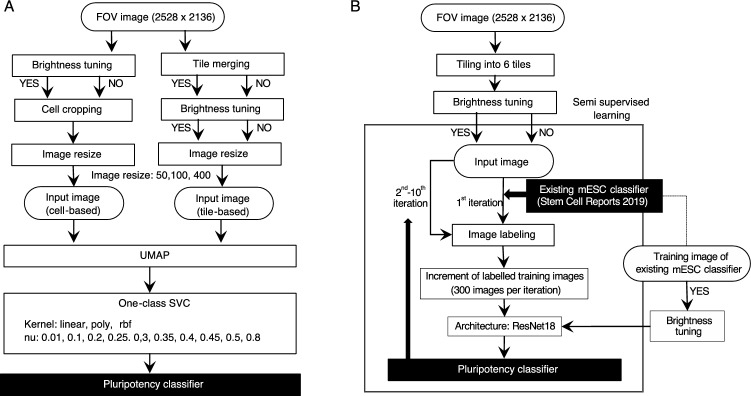
Modeling frameworks of image-based pluripotency assessment. (**A**) Outlier detection-based classification approach using the one-class SVC approach. (**B**) ResNet-based semi-supervised approach to train the classifier using an existing supervised pluripotency classification model of mouse ESCs.

Here, we proposed the use of two image AI frameworks to estimate distinct pluripotency signals from different molecular assays by classifying certain image units into “pluripotency or not,” without the label for each corresponding training image. First, as an unsupervised algorithm, a one-class SVC was trained on cropped cell images or tiled images to detect anomalous images, where we expected the detection ability of the models on single-cell level morphology changes or cell population-level differences, such as cell sparseness and distribution, respectively (Fig. 4A). In the one-class SVC model-building pipeline using cropped cell images as training data, all FOV images from all samples of all conditions first underwent brightness tuning and then cell cropping or directly underwent cell cropping without brightness tuning. In the pipeline using tiled images as training data, the images of four adjacent FOVs were merged into one tile image to accommodate more cells and avoid cell-free training images, or FOV images were used directly as tiled image units. Input images were created with and without brightness tuning of the tiled images. Cell- or tile-based images were resized into 50,400 pixels in each image unit to be used as input data for dimensionality reduction using UMAP in three dimensions. One-class SVC models were trained using the resulting dimensions with different kernels (linear, polynomial, and radial basis function) and various values of the ν parameter.

Second, we employed a deep-neural network architecture and incorporated a prebuilt classifier model reported by Waisman et al.^22^ as a guide model to label images in the initial round of semi-supervised model training iterations (Fig. 4B). The guide model was intended to classify undifferentiated and early differentiating mESCs at the early onset of differentiation stimuli by supervised training of ResNet-50 architecture, using transmitted light microscopy images of mESCs maintained in LIF + serum, and those induced for early differentiation of mesodermal cells. Importantly, the authors confirmed that the resulting classification model has remarkable predictability for human iPSCs in classifying images of undifferentiated and differentiated culture conditions. In this study, we retrained the model with the ResNet-50 architecture in our own environment using the original training image dataset provided by the authors, which contains 2,134 training images and 400 validation images from undifferentiated and differentiated mESCs in culture. In our semi-supervised pipeline, FOV images (2528 × 2136) were first resized into 1264 × 1068 and 6 images (640 × 480) were cropped from each resized image to unify the image size into the training image of the guide model. The merged tile images were then input into the iterative semi-supervised ResNet model, with or without brightness tuning. In the first iteration, all cropped images were labeled as undifferentiated and differentiated groups using the guide model. Among the pseudo-labeled images, the top 300 images with the highest class probability in the two groups were selected as the labeled dataset. The labeled dataset was used to update the ResNet-18 model and create a pseudo-labeled dataset. In the following iterations (iteration numbers 2–10), the top 300 pseudo-labeled images from the latest updated ResNet50 classifier were added to the labeled dataset in each iteration, and the dataset was used to update the ResNet50 model in the following iteration. The unlabeled dataset contained either images from this study (14,400 images) or images from this study and the original training data of the guide model (1600 images).

The predicted label for each input image unit for each sample was accumulated into the “pluripotency ratio for the sample,” which calculates the percentage of majority class in one-class SVC models and undifferentiated class in semi-supervised models. Unless otherwise noted, model predictions were made on FOV images at the last time point of image acquisition (i.e., time point 20 of Day 4), which was the closest time point to the following molecular experiments of pluripotency assessment.

### Model prediction, selection, and comparison with experimental molecular measurements

**Figure 5:**
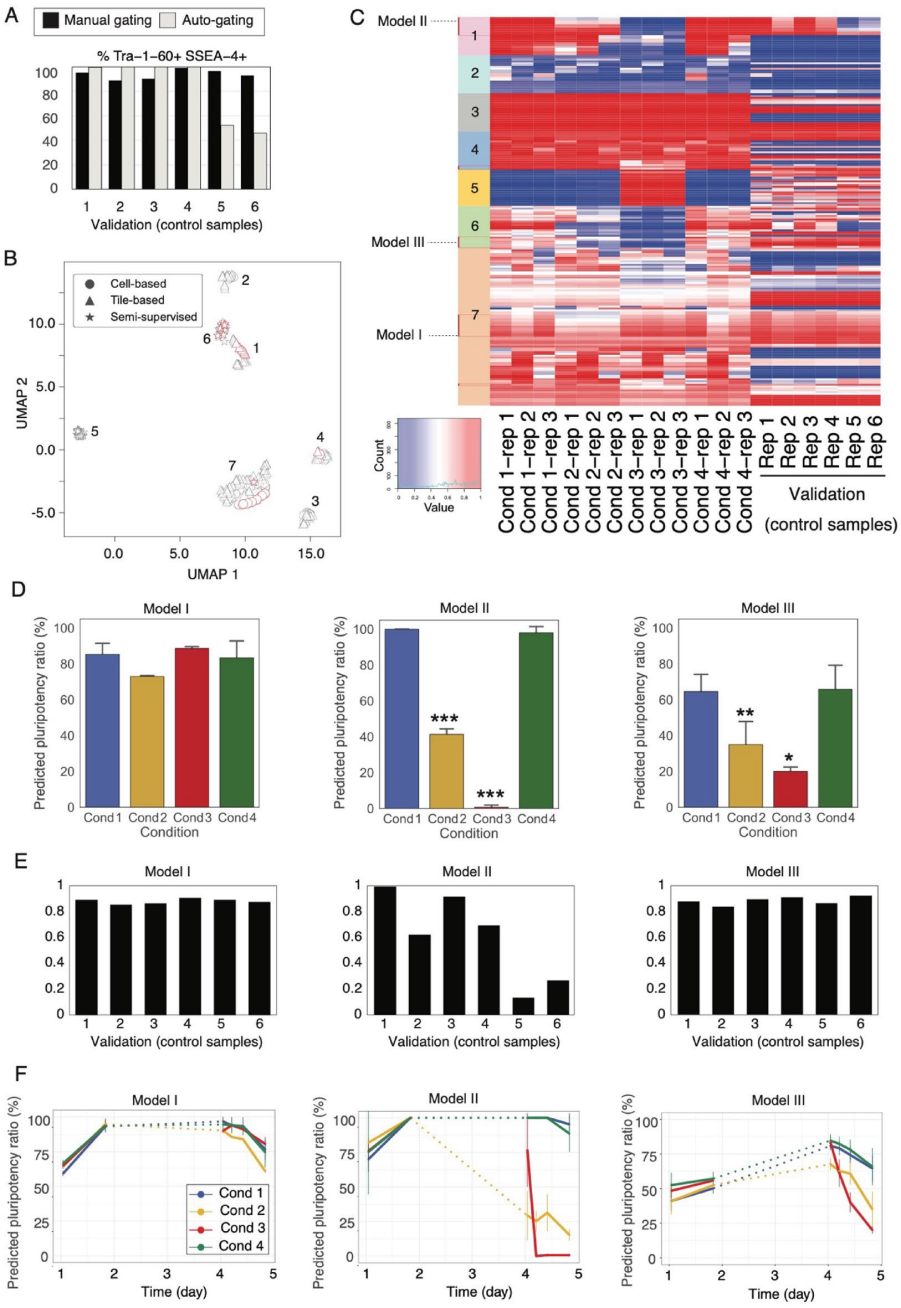
Model selection and prediction of pluripotency status. (**A**) FCM analysis of six control samples treated with the same procedure as Cond 1. Black and gray bars indicate FCM-derived pluripotency ratios (i.e., Tra-1–60 and SSEA-4 double-positive ratios) by manual- and auto-gating, respectively. (**B**) Model variations were mapped in the UMAP dimension using the model prediction results of pluripotency ratios of 12 samples (Cond 1–4). Model type (cell-based, tile-based one-class SVC, or semi-supervised framework) is indicated by the shape of the data points. The red frame around the data points indicates that the predictions of the highlighted models satisfy the selection criteria. (**C**) The model prediction results of pluripotency ratios for Cond 1–4 and the six validation samples of control pluripotency conditions are represented as a heatmap **(**blue to red indicate low to high predicted pluripotency ratio). The color-coded panel besides each model indicates the clusters seen in Fig. 5C, and the cluster numbers correspond in both figures. The best-fit models selected by correlation with molecular pluripotency markers (Models I–III) are indicated. (**D**–**E**) Predicted pluripotency ratio of the selected three models for the four distinct conditions (D) and the validation 6 samples for control pluripotency conditions (E). (**F**) Time-series prediction of pluripotency ratio on the images of the first and the last time points of Day 1 and timepoints 1, 5, 10, and 20 (last) of Day 4.

The prediction results of the models with different frameworks, preprocessing steps, and parameters were compared with the experimental molecular measurements. To validate the consistency of the prediction ability for different experimental batches, we performed a cell culture assay with six replicates using control pluripotency conditions as Cond 1, followed by FCM analysis (Fig. 5A). Results of manual- and auto-gating-based FCM analysis, shown as black and gray bars, respectively, present distinct patterns in FCM-derived pluripotency ratios (Supplementary Fig. 4). Notably, replicates 5 and 6 showed lower pluripotency ratios using auto-gating. Intersample changes were moderate in FCM data with manual-gating and no visible differences were detected by the experts’ visual inspection for all six replicates.

The model variations predicting pluripotency ratios for 12 samples under four distinct conditions with three replicates were mapped to the UMAP representation in Fig. 5B and are shown in the heatmap in Fig. 5C. Clear model clusters (clusters 1–7) emerged, depending mostly on the model framework. Models thought to reflect maintenance of pluripotency were highlighted with a red frame in the UMAP dimensions based on the following criteria: (1) the average predicted pluripotency ratio in Cond 1 and six validation control pluripotency samples was maintained at > 60% in each dataset and (2) sample-based variance value of predicted pluripotency ratio among all samples in Cond 1–4 was clearly detected as > 50. Based on the rationale that a model with sufficient predictive power for practical use might be robust to small differences in parameters, we identified three clusters (clusters 1, 6, and 7) in which models meeting these criteria had various parameter variations. The model variations that met the conditions within these clusters corresponded to cell- and tile-based one-class SVC (clusters 7 and 1) or a semi-supervised modeling framework (Cluster 6). The best-fit model for the molecular assessment of pluripotency was selected from each of the selected clusters based on the Pearson’s correlation of the predicted pluripotency ratio and immunostaining results (Supplementary Fig. 5). Notably, the correlations were calculated for the average value of each of the four conditions.

The predictions of the three resulting models for the four distinct conditions are shown in Fig. 5D**,** and those for the validation of the six samples are shown in Fig. 5E. Model I, with a cell-based one-class SVC framework, selected from Cluster 7, had the highest correlation with Oct4 with manual thresholding (*r* = 0.998), which was also highly correlated with Oct4 gene measurements using qPCR and RNA-Seq. Model I displayed a high correlation with surface marker protein measurements by FCM with auto-gating (*r* = 0.912). Model II, from Cluster 1 and with the tile-based one-class SVC framework, had the highest correlation with Nanog measured by immunostaining with automated thresholding (*r* = 0.961), which also correlated with Nanog gene expression values. Model III, selected from Cluster 6, which is a collection of models with the semi-supervised framework, correlated highly with the expression of Nanog protein (immunostaining with automated threshold; *r* = 0.978) and Nanog gene. Models II and III exhibited high correlation with surface marker proteins measured by FCM with manual-gating (*r* = 0.908 and 0.938, respectively).

In contrast to the validation control pluripotency samples, where each sample is associated with a matching image, Model I was highly correlated with surface marker protein measurements with manual-gating (*r* = 0.990, *r*^2^ = 0.980) and Model II was highly correlated with the same data as the auto-gating results (*r* = 0.901, *r*^2^ = 0.811). However, Model III did not have the predictability of FCM data with either gating strategy. This may be because the guide model referenced in the semi-supervised framework in Model III was built on top of the images of differentiation and undifferentiation conditions rather than heterogeneity under controlled pluripotency conditions. There were noteworthy differences between automated and manual thresholds in the quantification of protein intensities of immunostaining and FCM data, which are often debated^24^. For the immunostaining data, BASC-based automated thresholding of Nanog protein intensity was highly correlated with the gene expression profiles measured by RNA-Seq and qPCR. In contrast, the Oct4 protein had a high correlation with its gene expression profile when the intensity threshold was set manually at the valley of the bimodal peak. Although the robustness of quantification was not within the scope of this study, it is interesting to note that the unsupervised models that best-fit Nanog or Oct4 predicted automatic or manually gated FCM-based pluripotency ratios.

### Time-series prediction of Pluripotency

We applied the best models to images obtained at earlier time points to predict the onset of changes in pluripotent states represented in each model (Fig. 5F). For all of the three selected models, the pluripotency ratio was increased after the culture starts and plateaued by the last time point of Day 1. In addition, the predictions for replicates in each condition were relatively consistent, indicating that the models reliably captured the biological consequences reflected in the images. In Model I, which correlated with the Oct4 profile, the decrease in pluripotency ratio in all conditions became clear as late as day 4-time point 5, and a steep decline in Cond 2 was observed toward the last time point. Model II, which predicted the Nanog profile, identified a noticeable change in cellular states at the start of Day 4 in Cond 2, where the cells were stimulated by inactivated media from Day 2. Similarly, the changes in cellular states in Cond 2 in Model III were detectable at the onset of second-image capture. Conversely, for Cond 3, Models II and III detected extremely rapid changes in the cellular state immediately after stimulation by FBS on Day 4. For Cond 3, it is conceivable that Models II and III identify fluctuations, similar to those visually observable by humans, such as cell flattening and rapid increase in confluency over time. For Cond 1 and Cond 4, neither the model detected significant differences in protein and gene expression, nor were there significant fluctuations in cellular states observed. Notably, in Model II, drastic changes in the predicted pluripotency ratio were observed at an earlier onset in Cond 2 and Cond 3; thus, Model II may have the best potential to detect abnormalities in PSCs.

### Model prediction robustness

**Figure 6:**
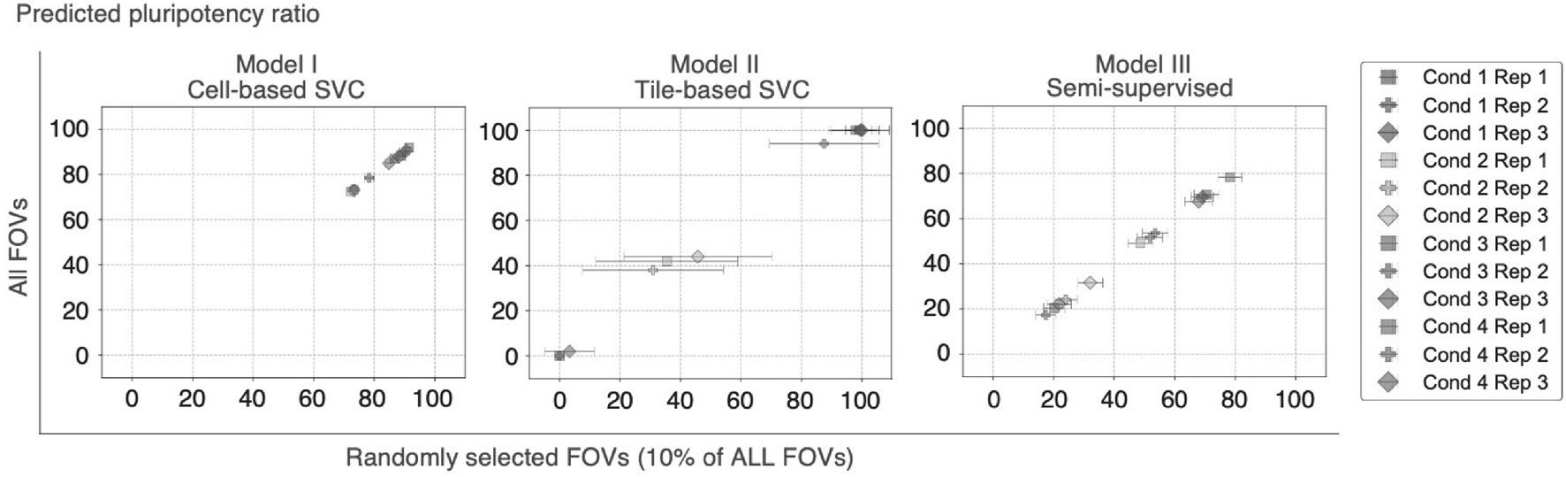
Robustness in pluripotency ratio prediction of the selected models for FOV selection. The X-axis shows mean and standard deviation of 100-times repeated predicted pluripotency ratios using 10% randomly selected FOVs. The Y-axis shows the predicted pluripotency ratio of each model using all FOVs of each sample.

To examine the robustness of the model prediction to the position within the microscopic field captured in the input images, we randomly sampled 10% of the files from all FOVs in each sample 100 times for model input and compared the predicted pluripotency ratio with the prediction using all FOVs as input images (Fig. 6). The mean pluripotency ratio among the 100 samples was consistent with the predicted value for all images (Pearson’s correlations and regression coefficients were > 0.99 for all three models). In contrast, the deviations of random inputs were larger in Model II (tile-based one-class SVC model) but significantly smaller in Model I (cell-based one-class SVC model). This may be due to the larger size, and thus, the smaller number of input images in Model II, which takes merged tiles, and the smaller size and large number of input images in Model I, which takes individual cell images.

We have also compared the average Oct4 and Nanog intensity for each FOV from the immunostaining data for all samples and the model prediction (Model III-Tile-based unsupervised model) which uses tiles in the FOV as an input (Supplementary Fig. 6). The results showed a good correlation, especially in Nanog, between actual averaged intensity values and predicted distance from the model’s origin, although the model has not learnt any molecular information nor the differentiation/undifferentiation labels.

Finally, the applicability of the models was tested using three different iPS cell lines, 1231A3, ND50018 and ND50019 cultured in control conditions in the same well-plate settings with the validation study in Fig. 5a, and the cells were assessed by qPCR (*POU5F1* and *NANOG*) and flow cytometry (SSEA-4 and Tra-1-60) analysis. The cell line 1231A3 was derived in the same institution (Center for iPS Cell Research and Application, Kyoto University, Japan) with 201B7, which was used throughout in this study, but the origin cell type is peripheral blood, which is different from fibroblast-derived 201B7. ND50018 and ND50019 were both derived in NIH Center for Regenerative Medicine (CRM) in USA, but the origin cell types are different—umbilical cord blood and fibroblast cells, respectively (Supplementary Table 6). Overall, the expressions of *POU5F1* and *NANOG* were not synchronized except for ND50019 (correlation coefficient r in 1231A3, ND50018, ND50019 were -0.71, 0.05 and 0.79, respectively), and the FCM results for automatic and manual gates also had different patterns (correlation coefficient r in 1231A3, ND50018, ND50019 were -0.54, 0.3 and -0.04, respectively), which makes generalization of the prediction performance more complex (Supplementary Table 7). Even under such circumstances, the Semi-supervised model (Model III) showed relatively good correlation with the Oct4 expression and FCM results (manual-gating) where the correlation coefficients were ranging from 0.56 to 0.73 (Oct4) and 0.47 to 0.78 (FCM). The average double positive (SSEA-4 + /Tra-1-60 +) rate for 1231A3 was commonly below 90% for auto-gated and manual-gated FCM analyses, which was also predicted by Model I (cell-based). and Model III. On the other hand, Model II (tile-based, unsupervised) predicted very low pluripotency ratios for other cell lines.

## Discussion

Changes in PSC status occur within hours to days, weeks, or longer, in a highly dynamic and multifaceted manner, which involves epigenetic, transcriptional, and metabolic changes. Therefore, a single marker, assay, and technique may miss abnormal PSCs^25–27^. Consistently, in this study, frequently used molecular pluripotency markers and their assays showed distinct signals for pluripotency in iPSCs. We observed data discrepancies, particularly in the expression of the Oct3/4 gene and protein (Fig. 3). Statistical analyses of diffuse large B-cell lymphomas have shown that significant discrepancies can occur between the expression levels of RNAs and their corresponding proteins^28^. These discrepancies may reflect inherent differences in processes from transcription to protein synthesis. Moreover, variations in the results suggest that relying solely on gene or protein expression to detect changes in the cellular status may be insufficient. Our methodology, which allows for independent model optimization for gene and protein expression, can detect changes in cellular states in both scenarios, in the face of such discrepancies. Alternatively, each model can be optimized as a comprehensive signal detection method that captures cellular states reflecting various molecular markers. Regarding the application of the predictive model to temporal changes in cellular states observed in Fig. 5F, these models demonstrate that variations in cellular states can be identified through noninvasive image analysis > 24 h before invasive testing is required, for example in cell production. These predictive models may provide significant advantages for monitoring cellular conditions during manufacturing in closed environments where invasive testing is difficult, and may allow for process control, such as stopping a process when an abnormality occurs.

Our methodology offers several advantages: it enables noninvasive detection, obviates the need for pre-labeling, enables detection at the earliest developmental stage, and enables research into the interrelationships and sequences of molecular and morphological changes in pluripotent cells. These benefits are particularly impactful in regenerative medicine manufacturing, allowing continuous monitoring of cell culture and quality assessment based on protein and gene expression without cell destruction or introduction of potential contaminants. This approach not only maintains cell integrity and viability for therapeutic applications but also opens new avenues for research in the clinical application of PSCs, offering a novel perspective on the dynamics of pluripotency.

Additionally, this could provide biological insights. Oct4 expression levels were significantly decreased only in Cond 2, using multiple measurement methods. This profile was well predicted by models that use individual cell images as input (cell-based one-class SVC), which may indicate that the change in visual signals in Cond 2 occurred at the single-cell level. Interestingly, several pathways related to cell morphology regulation were significantly upregulated in Cond 2 compared to those in Cond 1. One of the significantly upregulated genes in Cond 2 is involved in the regulation of cell–cell adhesion, CITED2, which directly regulates Oct4 expression in ESCs^29^. The same pathway includes GLI3, which was upregulated in Cond 2, and the relationship between the gene and Oct4, as well as morphological changes, were suggested to be present in cancer stem cells^30^. In contrast, Nanog expression decreased in Cond 2 and Cond 3 but was more significant in Cond 3, in which the cell number homeostasis pathway was significantly downregulated. We obtained the best-fit models for this profile based on tile-level input images. Together with the fact that the experts’ visual inspection could only detect visual changes in Cond 3, the models may detect the population-level dynamics of the cells, which may be reflected in the changes in confluence and cell density.

In this study, we developed a model to predict the expression of markers that reflect an undifferentiated state in cell images. This suggests that predicting the variability in the expression of other intracellular molecules may be possible, as long as their expression levels influence the appearance of cells and/or population-level dynamics such as confluency. If our approach could be expanded to create models that predict variability in the expression of an intracellular molecule by conforming to the conditions outlined, not only would it contribute to cell research, but its application in the manufacturing of cell products would also enable advanced evaluation of cell quality, moving beyond mere assessment of the undifferentiated state. In recent years, a bioinformatics pipeline of pluripotent cell profiling has been applied to evaluate the holistic similarity and dissimilarity of the transcriptome profile compared with that of a large set of somatic and pluripotent samples^31,32^. It would be instructive to extend our modeling strategy to predict signals from these empirical approaches. Moreover, integration of the model-building workflow in this study and the new modality of image technologies, such as computational holographic imaging or the RM-DIC system, realizing the resolution of subcellular structures in a live cell^33,34^ may capture a novel transient state of pluripotency at early time points, and thus providing a robust and infallible pipeline for regenerative medicine. We successfully predicted intracellular molecular dynamics from images of the 201B7 iPSC line, marking a significant advancement in understanding cellular processes. However, the generalizability of our model to other stem cell types, including various iPS and embryonic stem (ES) cell lines, remains an open question. This limitation underscores the necessity for broader data collection efforts across a diverse range of stem cell types. Such limitation was reflected in the low predictability of Model II in different cell lines. This may indicate that Model II needs per-cell type calibration as the field of coverage is larger in the input image of this model and the confluence and cell density were different amongst different cell lines. Amongst the tested cell lines, ND50019 showed better correlation between model prediction and the destructive assessments. Another possible hypothesis is based on the commonality of origin cell type of the iPS cell lines between training and test data, which requires further comprehensive investigation.

Moreover, it is crucial to consider the genetic stability of the cell lines used. Previous research has shown that genetic aberrations, such as mutations in key regulatory genes, can lead to the sustained expression of pluripotency markers like Oct4 and Nanog, even in differentiated cells^35^. Therefore, comprehensive genetic screening is essential to rule out such aberrations and confirm the reliability of our findings. Techniques such as whole-genome sequencing and comparative genomic hybridization can be employed to detect these abnormalities^36^. By incorporating these genetic screening methods, we can ensure that our AI model accurately reflects the pluripotent state and differentiation potential of the cells, thus strengthening the validity of our conclusions. By employing the methodology in this research, it is conceivable to develop comprehensive and adaptable predictive models for intracellular molecules. Such models would not only enhance our grasp of fundamental biological mechanisms but also potentially facilitate the advancement of regenerative medicine and disease modeling. Future studies should, therefore, focus on expanding the dataset and refining the model to improve its applicability across different cell lines, thereby increasing its utility in biological research and manufacturing regenerative medicine.

### Supplementary Information

Supplementary Information 1.

Supplementary Information 2.

## Data Availability

All the results from the experimental data including FCM analysis, immunostaining, qPCR and viability assessments were shown in Supplementary Table 4. The RNA-Seq dataset is available at GEO with accession number GSE256303. The prediction dashboard including image preprocessing and the selected prediction models is available as a web application upon request to the authors. The sample image data for the models is provided in https://github.com/TakeshiHase/sample-images-of-iPSCs.
