# Supplementary figures and images for: Noninvasive, label-free image approaches to predict multimodal molecular markers in pluripotency assessment
Source: Sci Rep. 2024 Jul 9;14:15760. doi: 10.1038/s41598-024-66591-z (PMC11231322; doi:10.1038/s41598-024-66591-z)

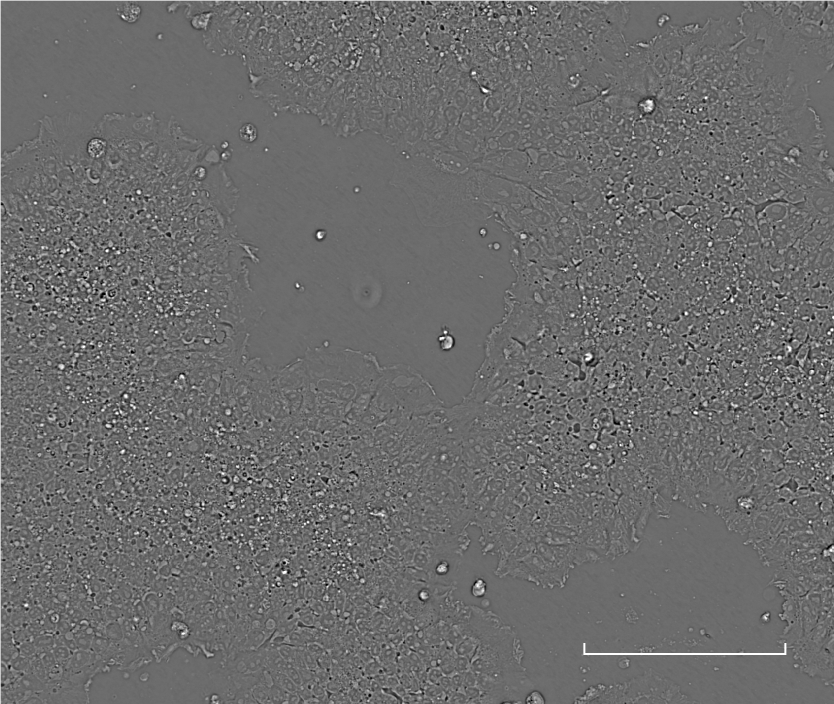

Supplement: Supplementary file 1 — Supplementary Information 1. [file 41598_2024_66591_MOESM1_ESM.zip › DPC_size bar/cond3_rep3.png]

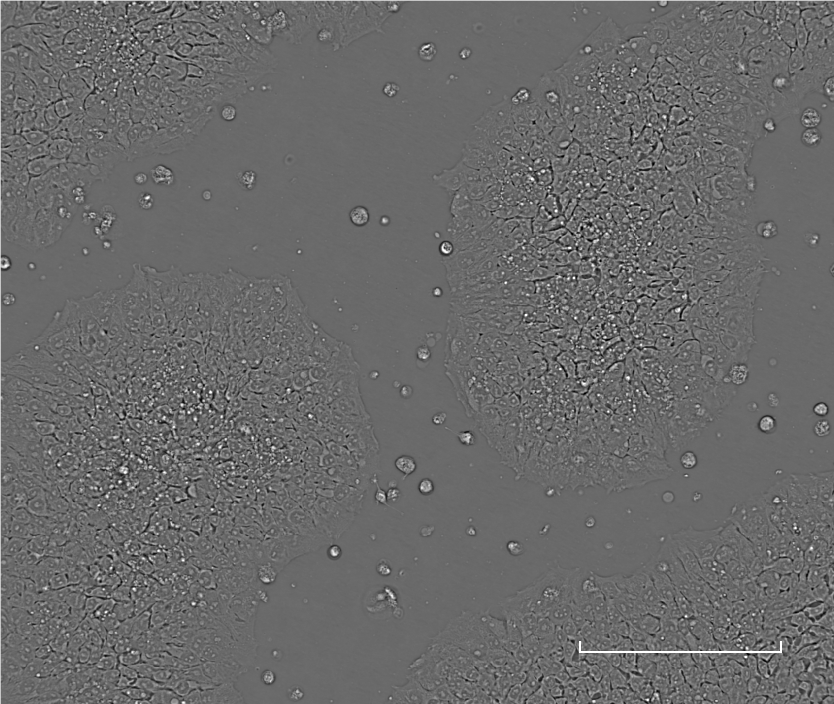

Supplement: Supplementary file 1 — Supplementary Information 1. [file 41598_2024_66591_MOESM1_ESM.zip › DPC_size bar/cond4_rep2.png]

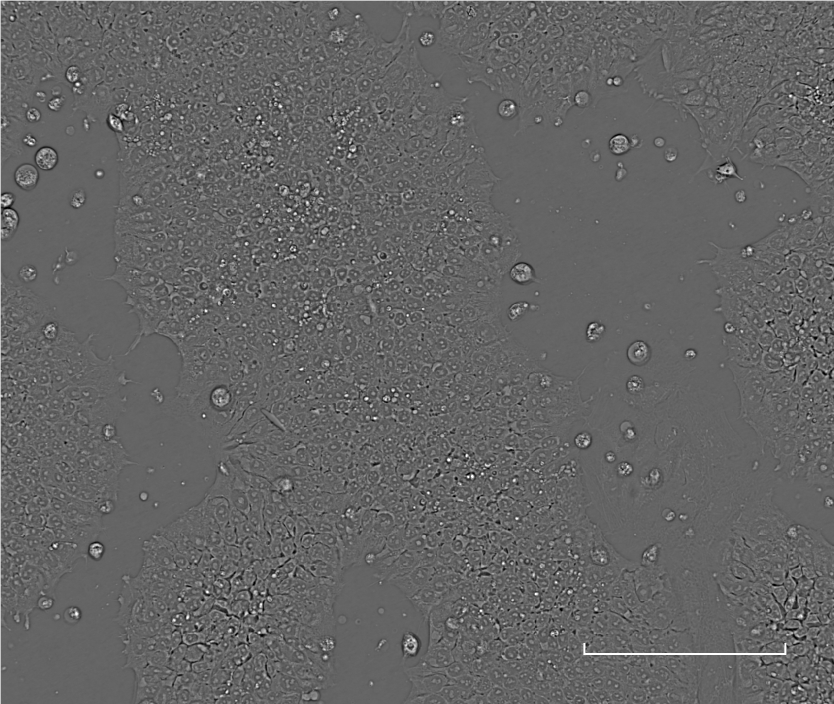

Supplement: Supplementary file 1 — Supplementary Information 1. [file 41598_2024_66591_MOESM1_ESM.zip › DPC_size bar/cond2_rep3.png]

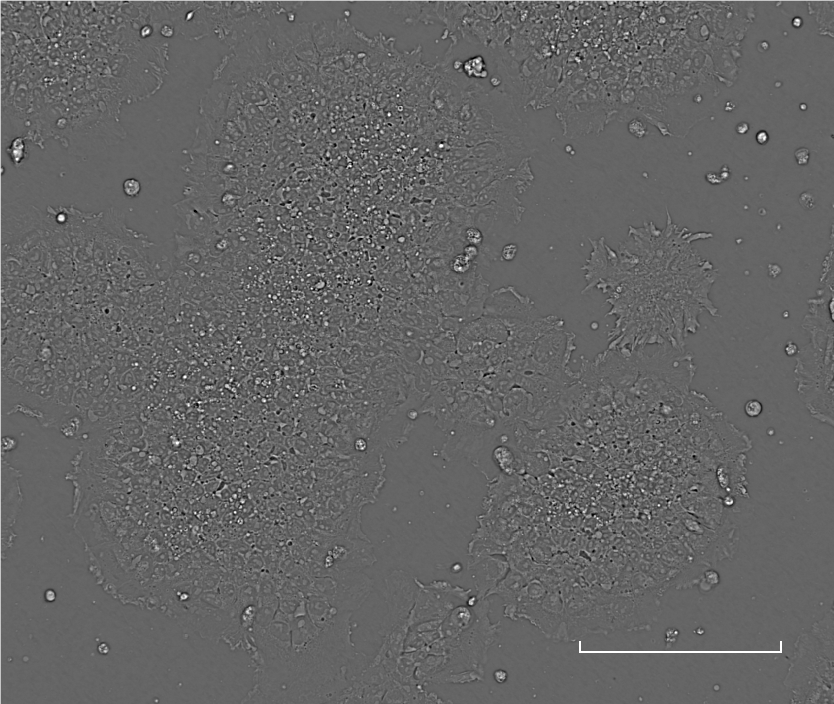

Supplement: Supplementary file 1 — Supplementary Information 1. [file 41598_2024_66591_MOESM1_ESM.zip › DPC_size bar/cond3_rep2.png]

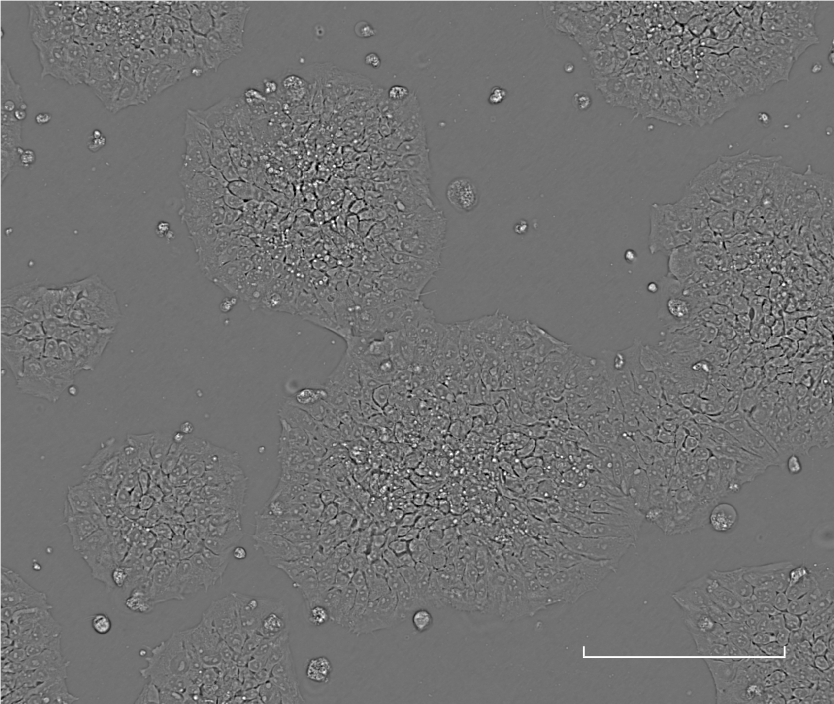

Supplement: Supplementary file 1 — Supplementary Information 1. [file 41598_2024_66591_MOESM1_ESM.zip › DPC_size bar/cond1_rep1.png]

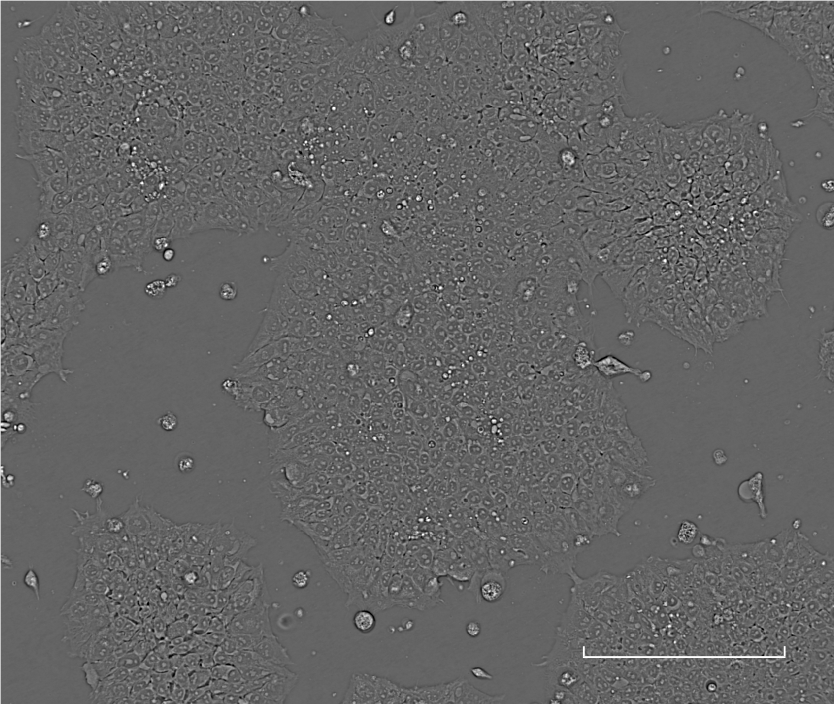

Supplement: Supplementary file 1 — Supplementary Information 1. [file 41598_2024_66591_MOESM1_ESM.zip › DPC_size bar/cond2_rep1.png]

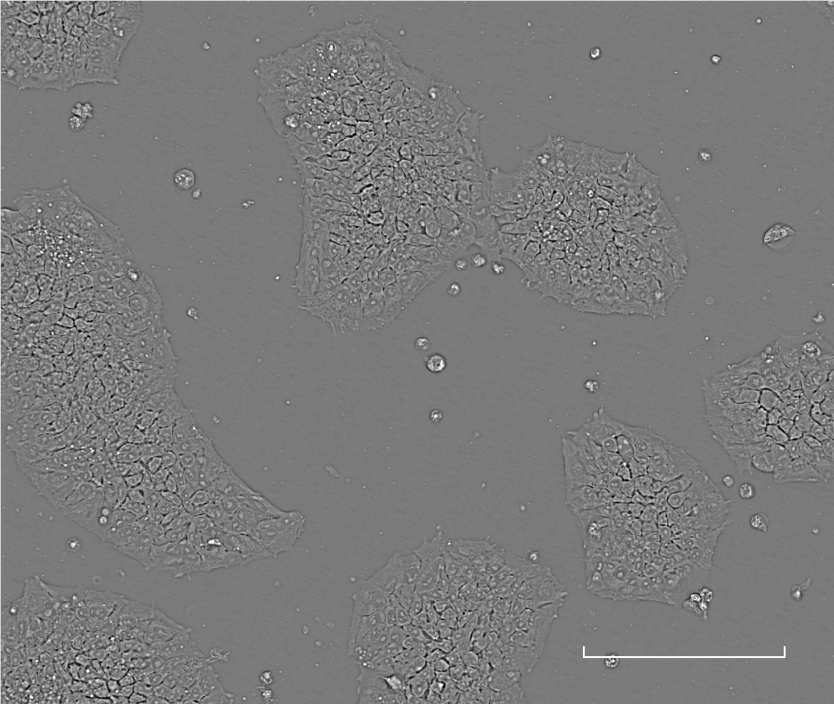

Supplement: Supplementary file 1 — Supplementary Information 1. [file 41598_2024_66591_MOESM1_ESM.zip › DPC_size bar/cond4_rep1.png]

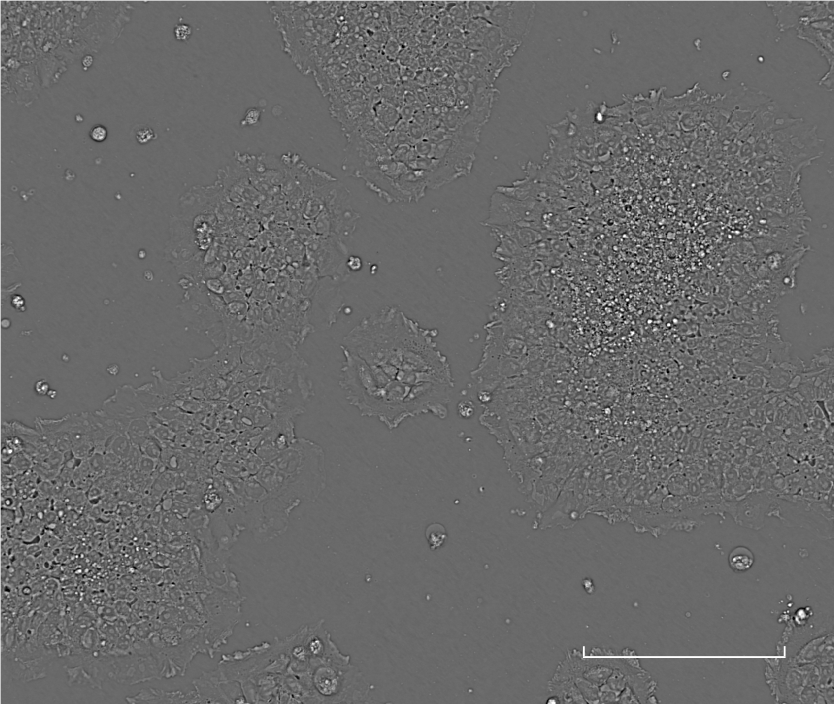

Supplement: Supplementary file 1 — Supplementary Information 1. [file 41598_2024_66591_MOESM1_ESM.zip › DPC_size bar/cond3_rep1.png]

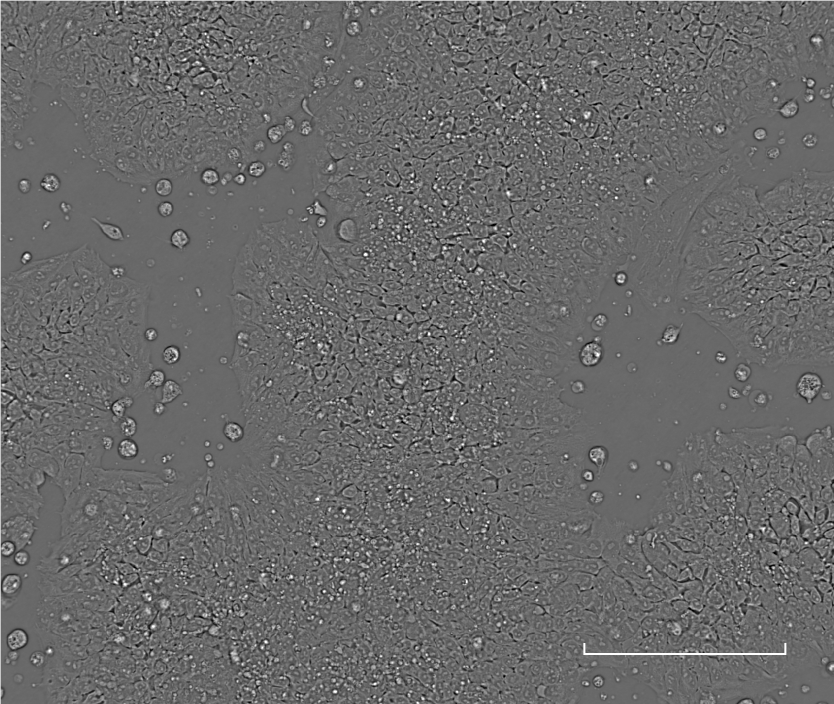

Supplement: Supplementary file 1 — Supplementary Information 1. [file 41598_2024_66591_MOESM1_ESM.zip › DPC_size bar/cond1_rep3.png]

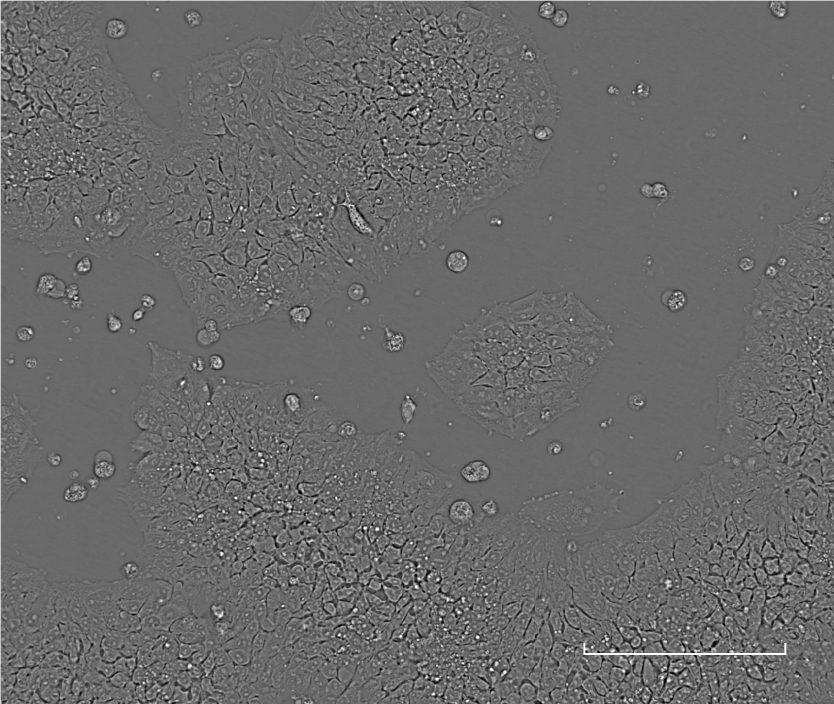

Supplement: Supplementary file 1 — Supplementary Information 1. [file 41598_2024_66591_MOESM1_ESM.zip › DPC_size bar/cond4_rep3.png]

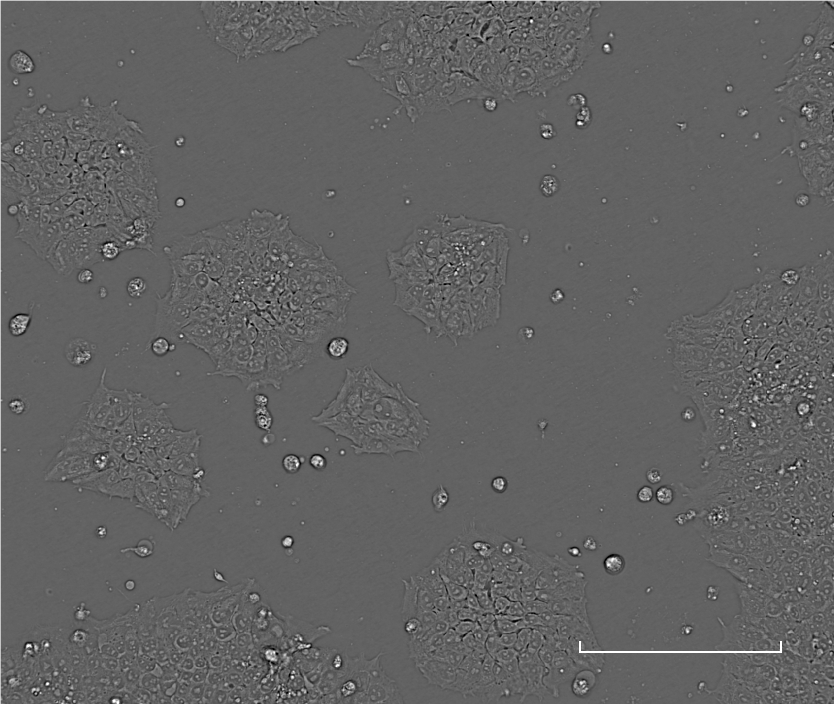

Supplement: Supplementary file 1 — Supplementary Information 1. [file 41598_2024_66591_MOESM1_ESM.zip › DPC_size bar/cond2_rep2.png]

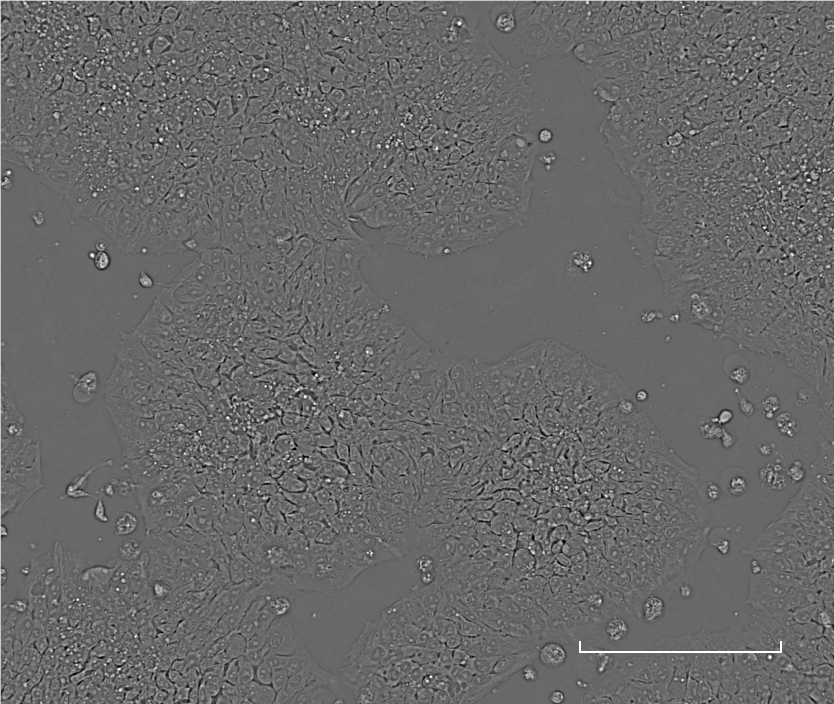

Supplement: Supplementary file 1 — Supplementary Information 1. [file 41598_2024_66591_MOESM1_ESM.zip › DPC_size bar/cond1_rep2.png]
