# Supplementary material for: Noninvasive, label-free image approaches to predict multimodal molecular markers in pluripotency assessment
Source: Sci Rep. 2024 Jul 9;14:15760. doi: 10.1038/s41598-024-66591-z (PMC11231322; doi:10.1038/s41598-024-66591-z)

Supplementary Information

for

Ryutaro Akiyoshi and Takeshi Hase et al.

Noninvasive, Label-free Image Approaches to Predict Multimodal Molecular Markers in Pluripotency Assessment

Supplementary Tables

Supplementary Table 1

Antibodies used for flow cytometry.

Target Antigen	Host/Isotype	Dilution
FITC-TRA-1-60	Mouse/IgM	1:20
SSEA-4	Mouse/IgG3	1:10

Supplementary Table 2

(a) Primary antibodies for immunochemistry

Target Antigen	Host/Isotype	Dilution
Oct-3/4	Mouse/IgG2b	1:100
NANOG	Mouse/IgG2aκ	1:500

(b) Secondary antibodies for immunochemistry

Host/Target Antigen	Marker	Dilution
Donkey/Mouse IgG(H+L)488	Alexa488	1:1000
Hoechst33342	-	1:5000

Supplementary Table 3

(a) Primers and probes for RT-qPCR

Gene	Assay ID
ACTB	Hs01060665_g1
POU5F1	Hs04260367_gH
NANOG	Hs02387400_g1

(b) Thermal-cycling conditions for RT-qPCR

Cycle	1cycle		PCR (40cycles)	
Step	UNG incubation	Polymerase activation	Denature	Anneal/Extend
Temperature	50°C	95°C	95°C	60°C
Time	2min	20sec	1sec	20sec

Supplementary Table 4

Molecular assessment results for pluripotency markers and cell viability

(a) FCM (% positive/live cells)

		Auto-gating (mindensity2)			Manual-gating			Auto-gating (tailgate)		
		$SSEA4^+$	$Tra1\text{-}60^+$	$SSEA4^+/Tra1\text{-}60^+$	$SSEA4^+$	$Tra1\text{-}60^+$	$SSEA4^+/Tra1\text{-}60^+$	$SSEA4^+$	$Tra1\text{-}60^+$	$SSEA4^+/Tra1\text{-}60^+$
Cond1	Rep 1	100.0	100.0	100.0	100.0	96.5	95.7	99.9	100.0	99.9
	Rep 2	99.9	98.7	98.7	100.0	96.0	95.1	98.8	99.9	98.8
	Rep 3	100.0	99.1	99.1	100.0	94.1	93.1	99.3	99.5	99.0
Cond2	Rep 1	100.0	100.0	100.0	100.0	90.3	88.4	99.9	100.0	99.9
	Rep 2	99.2	91.0	91.0	100.0	96.3	95.3	74.1	90.2	73.9
	Rep 3	96.0	81.3	81.3	100.0	92.4	91.7	66.4	78.5	65.4
Cond3	Rep 1	100.0	99.3	99.3	100.0	93.7	92.1	99.8	99.8	99.6
	Rep 2	100.0	97.8	97.8	100.0	94.0	92.4	99.5	99.8	99.4
	Rep 3	98.4	100.0	98.3	100.0	93.6	92.0	98.1	99.7	98.0
Cond4	Rep 1	100.0	99.7	99.7	100.0	93.8	92.3	99.7	99.8	99.5
	Rep 2	99.8	99.9	99.8	100.0	97.2	96.5	99.9	99.7	99.7
	Rep 3	100.0	98.8	98.8	100.0	95.7	94.9	90.6	99.3	90.5

(b) Immunochemistry (% positive cells)

		Auto-threshold		Manual-threshold	
		$Oct4^+$	$Nanog^+$	$Oct4^+$	$Nanog^+$
Cond1	Rep 1	63.8	54.4	93.2	92.1
	Rep 2	75.4	52.5	92.8	92.5
	Rep 3	88.3	33.9	90.9	91.6
Cond2	Rep 1	10.1	18.9	73.6	73.6
	Rep 2	32.8	20.6	81.9	84.0
	Rep 3	55.0	17.1	81.0	85.3
Cond3	Rep 1	16.7	5.4	96.9	83.5
	Rep 2	51.0	9.9	96.6	92.4
	Rep 3	92.1	4.2	96.3	91.1
Cond4	Rep 1	62.4	66.6	92.7	92.8
	Rep 2	76.0	54.7	92.3	93.6
	Rep 3	86.1	59.9	89.0	90.1

(c) RT-qPCR (2^-ΔΔCt) and RNAseq

		RT-qPCR		RNAseq (FPKM)	
		Oct4	Nanog	Oct4	Nanog
Cond1	Rep 1	0.96	1.07	1.57	46.69
	Rep 2	1.06	1.07	1.03	47.23
	Rep 3	0.98	0.87	1.36	43.64
Cond2	Rep 1	0.58	0.36	0.41	20.22
	Rep 2	0.48	0.33	0.79	24.03
	Rep 3	0.48	0.35	0.58	24.56
Cond3	Rep 1	0.98	0.26	1.41	9.93
	Rep 2	0.93	0.25	1.14	11.35
	Rep 3	0.97	0.26	1.04	10.17
Cond4	Rep 1	1.04	1.02	1.32	44.60
	Rep 2	0.93	0.38	1.22	47.08
	Rep 3	0.9	0.63	1.03	48.14

(d) Cell viability (%)

		Method	
		ViCELL	FCM
Cond1	Rep 1	96.7	96.5
	Rep 2	90.4	98.0
	Rep 3	90.3	89.9
Cond2	Rep 1	96.2	97.3
	Rep 2	92.5	94.8
	Rep 3	94.6	79.1
Cond3	Rep 1	97.9	98.6
	Rep 2	95.5	96.8
	Rep 3	96.6	97.3
Cond4	Rep 1	91.9	96.7
	Rep 2	93.2	90.3
	Rep 3	89.9	86.8

(e) FCM (% positive/live cells) for validation pluripotency control samples (201B7)

	Auto-gating	Manual-gating
1	99.7	95.2
2	99.9	89.1
3	99.9	90.2
4	99.9	99.2
5	52.3	96.6
6	45.9	93

(f) FCM (% positive/live cells) and RT-qPCR(2^-ΔΔCt) for validation pluripotency control samples of other cell lines

Cell line: 1231A3

	FCM Auto-gating	FCM Manual-gating	RT-qPCR Oct4	RT-qPCR Nanog
1	99.48	78.60	0.924	1.008
2	99.86	71.50	0.492	1.073
3	99.66	75.20	0.689	1.072
4	45.43	92.00	1.323	0.949
5	99.97	82.80	1.407	0.903
6	24.04	80.10	1.714	1.006

Cell line: ND50018

	FCM Auto-gating	FCM Manual-gating	RT-qPCR Oct4	RT-qPCR Nanog
1	99.97	97.70	1.714	1.006
2	98.42	91.50	0.516	0.753
3	99.38	87.70	0.982	1.532
4	49.27	95.90	0.652	1.022
5	99.20	93.10	1.378	1.1
6	99.91	90.50	2.631	1.159

Cell line: ND50019

	FCM Auto-gating	FCM Manual-gating	RT-qPCR Oct4	RT-qPCR Nanog
1	99.92	98.20	0.663	0.892
2	99.89	91.40	0.795	0.867
3	99.89	96.40	0.8	0.739
4	99.59	99.90	1.175	1.065
5	98.80	99.60	1.141	1.35
6	99.96	99.20	1.767	1.218

Supplementary Table 5

Visual related pathways and differentially expressed genes.

Significantly up- (or down-) regulated genes from DEG analysis in Cond2 or Cond 3 against Cond1 with the criteria of log2FC > 1.0 (or -1.0) and adjusted P-value < 0.01 were shown for the pathways selected by association with cell morphology. We calculated enrichment score (adjusted p-values) for these pathways by using over-representation analysis which were shown in the parenthesis where ** and * indicate adjusted p-value < 0.1 and 0.05, respectively, while NS indicates non-significant. The differentially expressed genes in the significantly enriched morphology-related pathways at the pathway-level were marked on the volcano plot in Figure 3F.

ID	Description	genes up-regulated under cond2	genes down-regulated under cond2	genes up-regulated under cond3	genes dow-regulated nder cond3
GO:0007162	negative regulation of cell adhesion	(NS); EPHA4/EFNA5/GLI3/EPHB2/BMP4	(NS); FOXJ1/CD9/IL20RB	(NS); BMP2/PRKCD/EPHB2/LOXL3/RND1	(**); SPRY4/AKNA/CEBPB/THBS1/VEGFA/SERPINE1/PIK3R1/SOCS1
GO:0008360	regulation of cell shape	(NS); COCH/FERMT2/CCL2	(NS); KDR/RND3/P2RY1	(NS); PALM3/VIL1/RND1/ARHGAP18/CORO1A	(NS); CDC42EP1/RND3/VEGFA/P2RY1
GO:0008361	regulation of cell size	(NS); EFNA5/MAP2/MAP1B/EPHA7	(NS)	(NS); OLFM1/TRPV2/MAP2/FSTL4/WNT5A/NRCAM	(NS); FGF13/IFRD1/VEGFA
GO:0010769	regulation of cell morphogenesis involved in differentiation	(**); EPHA4/EFNA5/FERMT2/EPHB2	(NS)	(NS); CSPG5/EPHB2/DAB2	(NS); SPRY4
GO:0010770	positive regulation of cell morphogenesis involved in differentiation	(*); EPHA4/FERMT2/EPHB2	(NS)	(NS); CSPG5/EPHB2/DAB2	(NS)
GO:0010771	negative regulation of cell morphogenesis involved in differentiation	(NS); EFNA5	(NS)	(NS)	(NS); SPRY4

GO:0022407	regulation of cell-cell adhesion	(**); CITED2/EFNA5/GLI3/EFNB1/BMP4/PCDH8/EPHA7/BMP7/CCL2	(NS); FOXJ1/THY1/CD9/ETS1/TNFSF11/IL20RB	(NS); BMP2/PRKCD/PCDH8/DPP4/CAV1/WNT5A/LOXL3/ADAM8/CORO1A	(NS); THY1/AKNA/SOX4/CEBPB/VEGFA/ZP3/NODAL/SOCS1
GO:0022409	positive regulation of cell-cell adhesion	(NS); CITED2/GLI3/EFNB1/BMP7/CCL2	(NS); THY1/ETS1/TNFSF11	(NS); DPP4/CAV1/WNT5A/ADAM8/CORO1A	(NS); THY1/SOX4/ZP3/NODAL/SOCS1
GO:0022604	regulation of cell morphogenesis	(*); EPHA4/EFNA5/COCH/FERMT2/EPHB2/CCL2	(NS); KDR/RND3/P2RY1	(NS); PALM3/CSPG5/EPHB2/VIL1/WNT5A/DAB2/RND1/ARHGAP18/CORO1A	(NS); SPRY4/CDC42EP1/RND3/VEGFA/P2RY1
GO:0032956	regulation of actin cytoskeleton organization	(NS); SPTBN2/EFNA5/FERMT2/LIMCH1/CTNNA2	(NS); RND3	(NS); PRKCD/MTSS1/TEK/VIL1/RND1/LMOD1/SYNPO/ARHGAP18/TMOD2/CORO1A	(NS); HAX1/CDC42EP1/PDGFA/CCN2/RND3/PIK3R1
GO:0045785	positive regulation of cell adhesion	(NS); EPHA4/CITED2/FERMT2/GLI3/EFNB1/BMP7/CCL2	(NS); KDR/THY1/SPOCK2/ETS1/TNFSF11/PDGFB	(NS); CSPG5/TEK/DPP4/CAV1/WNT5A/DAB2/ADAM8/CORO1A/CHRD	(**); FUT1/THY1/PDGFB/SOX4/SFRP2/VEGFA/SPOCK2/ZP3/CCN1/NODAL/SOCS1
GO:0048872	homeostasis of number of cells	(NS); CITED2/BMP4/ID2	(*); MAFB/PLA2G2A/ETS1/FLVCR1/IL20RB	(NS); CXCL6/PIK3CD/ASXL1/SOX9/ID2/TNFAIP3/CORO1A	(**); TSC22D3/MAFB/SLC7A11/SOX4/PMAIP1/VEGFA/GCNT4/SKIL
GO:0051495	positive regulation of cytoskeleton organization	(NS); FERMT2/LIMCH1/MAP1B	(NS)	(NS); PDE4DIP/MTSS1/TEK/VIL1/LMOD1/SYNPO	(NS); HAX1/CDC42EP1/CCN2
GO:0098727	maintenance of cell number	(NS); HESX1/BMP7	(NS); NANOG	(NS); ZIC3/LBH/SOX9/PTN	(NS); NANOG/SOX4/MYC/NODAL

Supplementary Table 6

	Role in this study	Institution	Source cell type/tissue	donor
201B7	Used throughout in this study including model training and validation	Center for iPS Cell Research and Application (CiRA)	Fibroblast of skin	Female-36Y
1231A3	Validation	Center for iPS Cell Research and Application (CiRA)	Peripheral blood	Female 29Y
ND50018	Validation	NIH Center for Regenerative Medicine (CRM)	Umbilical cord blood	Female <1D
ND50019	Validation	NIH Center for Regenerative Medicine (CRM)	Fibroblast of foreskin	Male <1M
iPSC-FH2.1	The template model of Semi-supervised models was validated using this cell line in the original study (Waisman et al 2019)	Instituto de Neurociencias	Fibroblast of foreskin	Male (age unspecified)

Supplementary Table 7

The molecular assays and model prediction results (upper) and their correlations (lower) in control conditions in three different iPS cell lines. The RT-qPCR results and FCM results are shown in 2^-ΔΔCt vs. averaged control conditions (equivalent to Cond1) and percentage of SSEA-4+/Tra-1-60+ cells in live cells, respectively. The model prediction results represent the pluripotency ratio (%).

	qPCR Oct_4	qPCR Nanog	FCM auto-gating		FCM manual gating		MODEL I		MODEL III		MODEL II	
1231A3	0.92	1.01	99.48		78.6		91.78		88.96		99.00	
	0.49	1.07	99.86		71.5		85.55		81.88		44.25	
	0.69	1.07	99.66	average	75.2	average	87.52	average	90.54	average	75.50	average
	1.32	0.95	45.43	78.07	92	80.03	89.53	0.88	89.67	0.89	91.50	0.62
	1.41	0.90	99.97		82.8		90.28		87.88		10.00	
	1.71	1.01	24.04		80.1		84.11		92.08		48.75	
ND50018	1.71	1.01	99.97		97.7		98.02		99.50		0.00	
	0.52	0.75	98.42		91.5		90.29		96.58		10.00	
	0.98	1.53	99.38	average	87.7	average	95.55	average	96.42	average	1.00	average
	0.65	1.02	49.27	91.03	95.9	92.73	97.72	0.95	98.88	0.98	0.00	0.02
	1.38	1.10	99.2		93.1		93.53		97.88		0.00	
	2.63	1.16	99.91		90.5		97.50		99.96		0.00	
ND50019	0.66	0.89	99.92		98.2		90.28		97.21		37.00	
	0.80	0.87	99.89		91.4		85.84		89.58		15.75	
	0.80	0.74	99.89	average	96.4	average	90.41	average	88.54	average	1.00	average
	1.18	1.06	99.59	99.68	99.9	97.45	93.51	0.91	99.25	0.91	0.50	0.09
	1.14	1.35	98.8		99.6		88.62		94.96		2.00	
	1.77	1.22	99.96		99.2		95.40		98.54		0.00	

Correlation coef. (r)		1231A3	ND50018	ND50019
MODEL I	qPCR Oct_4	-0.05	0.52	0.73
	qPCR Nanog	-0.52	0.40	0.32
	FCM auto-gating	0.45	-0.34	0.22
	FCM manual gating	0.41	0.37	0.73
MODEL III	qPCR Oct_4	0.65	0.73	0.56
	qPCR Nanog	-0.26	-0.15	0.61
	FCM auto-gating	-0.55	-0.20	-0.13
	FCM manual gating	0.47	0.57	0.78

Supplementary Figures

Supplementary Figure 1. Auto gating-based (upper) and Manual-gating (lower) based FCM data analysis – TRA-1-60 and SSEA-4 double positive cell filtering.

Auto gating

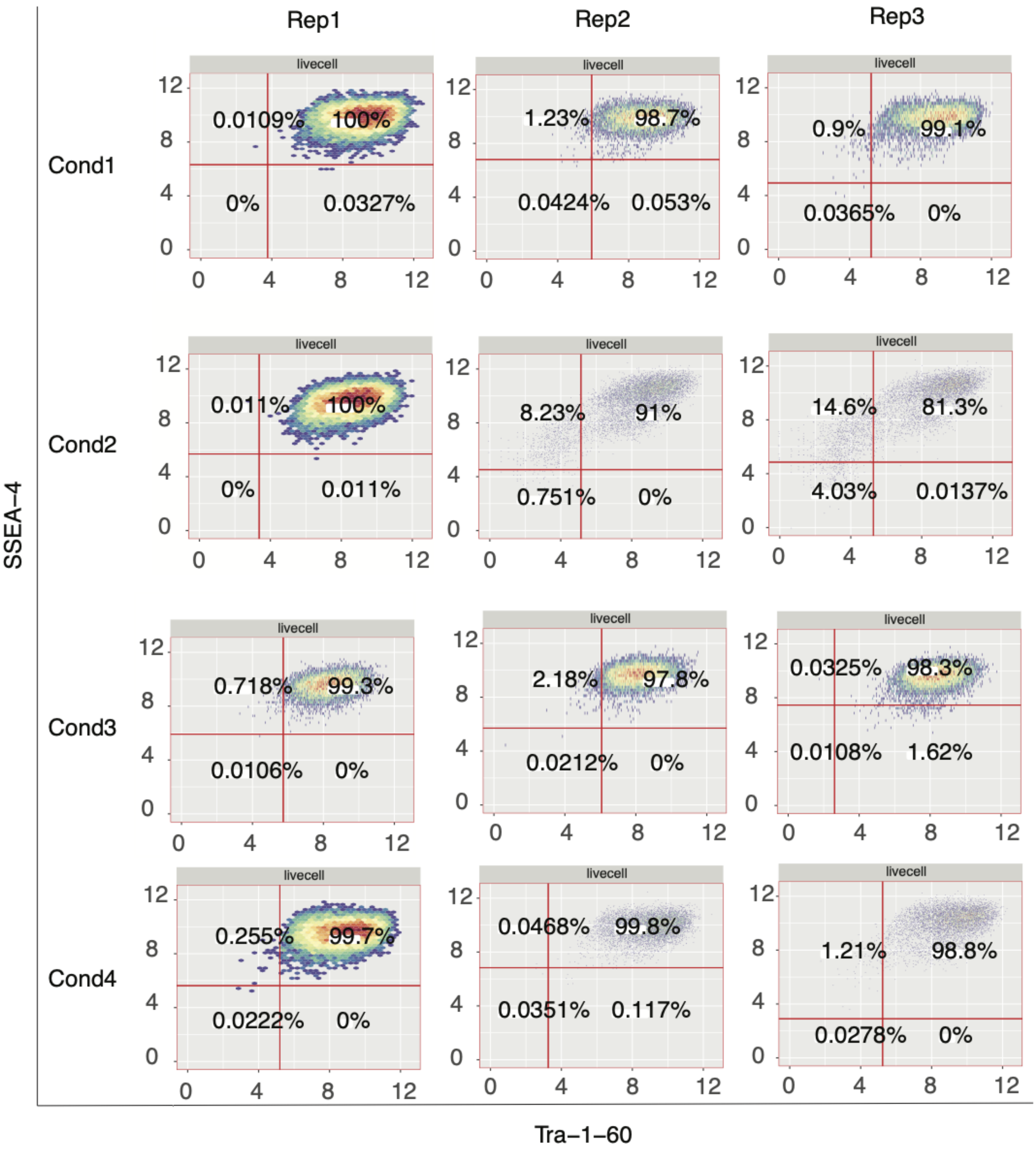

Manual gating

Cond1

Rep1

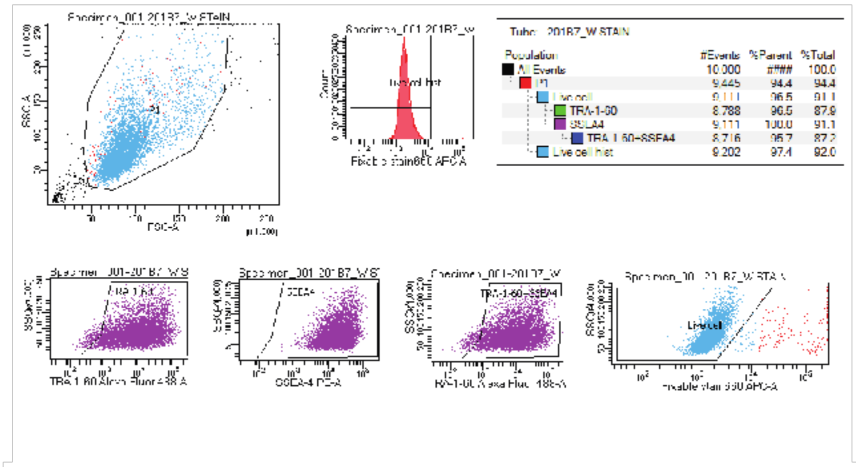

Rep2

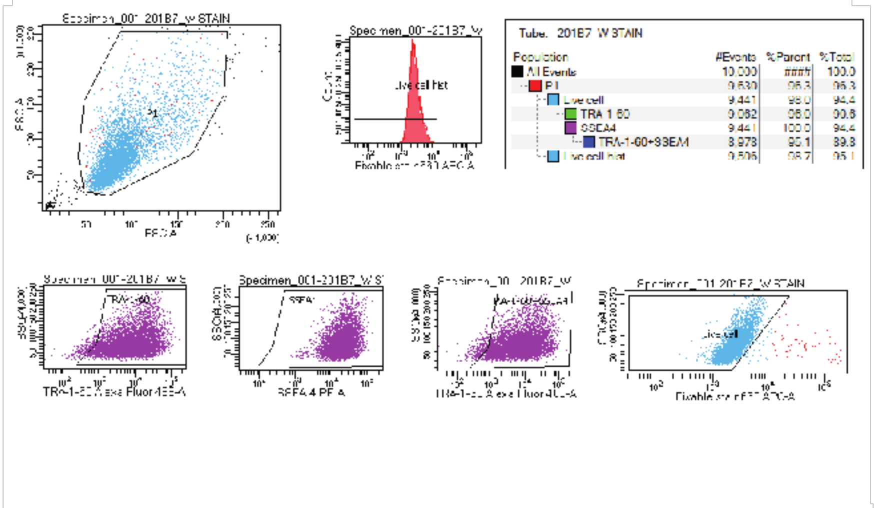

Rep3

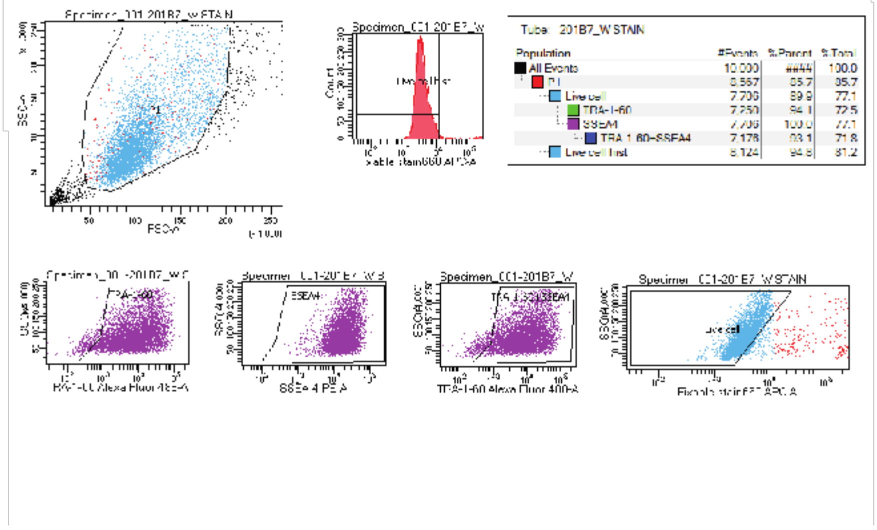

Cond2

Rep1

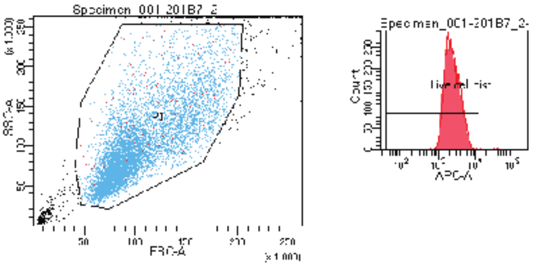

Tube: 201B7_2-1

Population	#Events	%Parent	%Total
All Events	10,000	####	100.0
P1	9,141	91.4	91.4
Live cell	8,890	97.3	88.9
TRA-1-60	8,026	90.3	80.3
SSEA4	8,890	100.0	88.9
TRA-1-60+SSEA4	7,863	88.4	78.6
Live cell hist	8,971	98.2	89.7

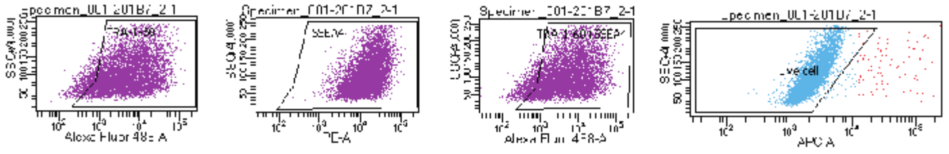

Rep2

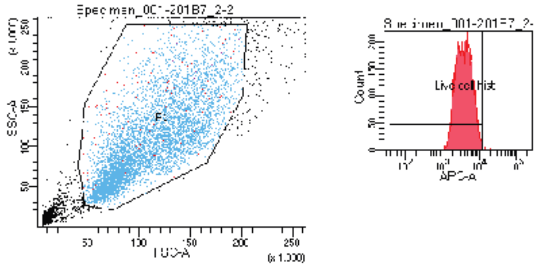

Tube: 201B7_2-2

Population	#Events	%Parent	%Total
All Events	10,000	####	100.0
P1	8,037	80.4	80.4
Live cell	7,619	94.8	76.2
TRA-1-60	7,335	96.3	73.4
SSEA4	7,619	100.0	76.2
TRA-1-60+SSEA4	7,263	95.3	72.6
Live cell hist	7,823	97.3	78.2

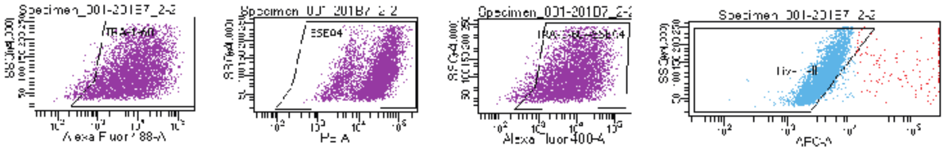

Rep3

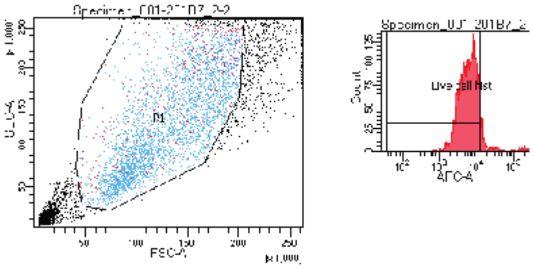

Tube: 201B7_2-2

Population	#Events	%Parent	%Total
All Events	10,000	####	100.0
P1	4,823	48.2	48.2
Live cell	3,813	79.1	38.1
TRA-1-60	3,524	92.4	35.2
SSEA4	3,813	100.0	38.1
TRA-1-60+SSEA4	3,496	91.7	35.0
Live cell hist	4,353	90.3	43.5

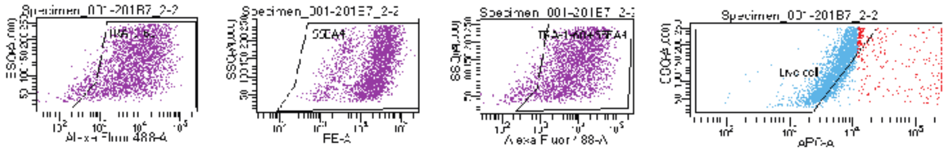

Cond3

Rep1

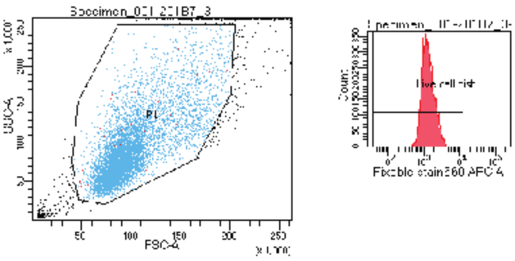

Tube: 201B7 3 1			
Population	#Events	%Parent	%Total
All Events	10,000	####	100.0
P1	9,297	93.0	93.0
Live cell	9,171	98.6	91.7
TRA-1-60	8,590	93.7	85.9
SSEA4	9,171	100.0	91.7
TRA 1 60+SSEA4	8,442	92.1	84.4
Live cell hist	9,224	99.2	92.2

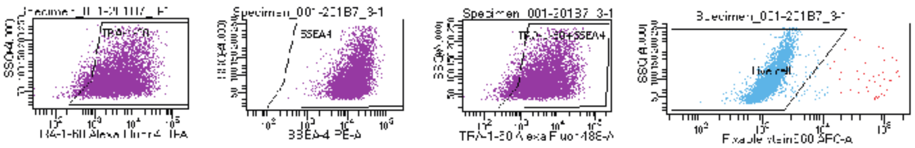

Rep2

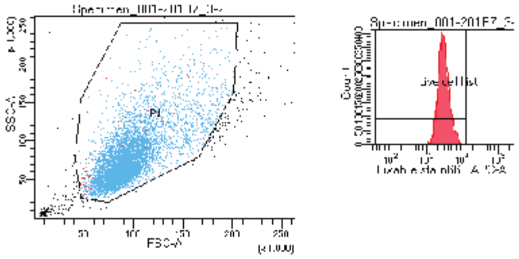

Tube: 201B7_3 2			
Population	#Events	%Parent	%Total
All Events	10,000	####	100.0
P1	9,556	95.6	95.6
Live cell	9,246	96.8	92.5
TRA 1 60	8,695	94.0	87.0
SSEA4	9,246	100.0	92.5
TRA-1-60+SSEA4	8,539	92.4	85.4
Live cell hist	9,418	98.6	94.2

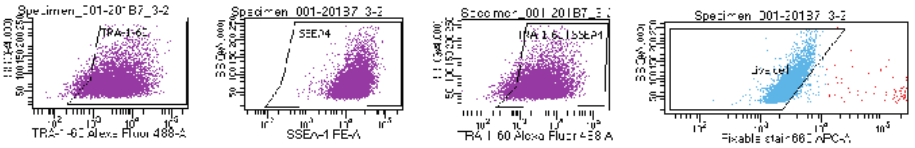

Rep3

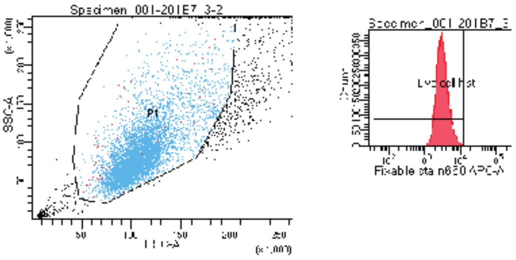

Tube: 201B7 3-2			
Population	#Events	%Parent	%Total
All Events	10,000	####	100.0
P1	8,989	89.9	89.9
Live cell	8,740	97.3	87.5
TRA-1-60	8,189	93.6	81.9
SSEA4	8,749	100.0	87.5
TRA-1-60+SSEA4	8,049	92.0	80.5
Live cell hist	8,886	98.9	88.9

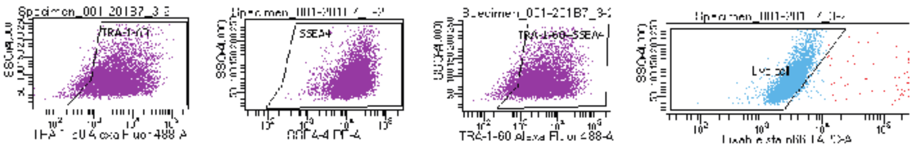

Cond4

Rep1

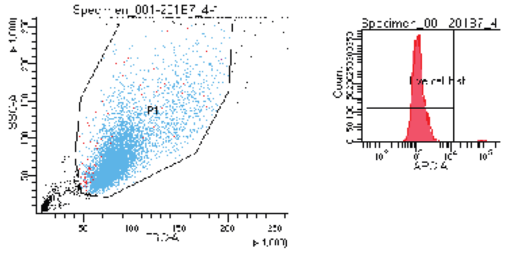

Tube: 201B7 4-1

Population	#Events	%Parent	%Total
All Events	10,000	####	100.0
P1	9,230	92.4	92.4
Live cell	8,934	96.7	89.3
TRA-1-60	8,381	93.8	83.8
SSEA4	8,934	100.0	89.3
TRA-1-60+SSEA4	8,250	92.3	82.5
Live cell hist	9,005	97.5	90.0

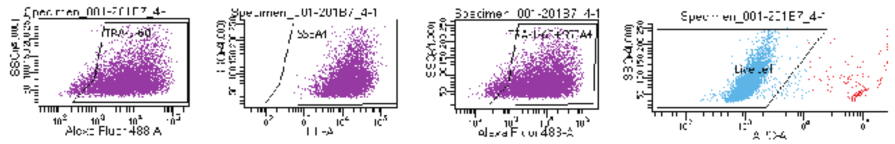

Rep2

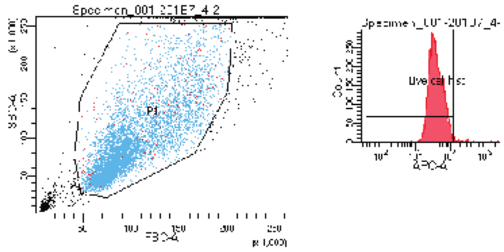

Tube: 201B7_4-2

Population	#Events	%Parent	%Total
All Events	10.000	####	100.0
P1	8.848	88.5	88.5
Live cell	7.989	90.3	79.9
TRA 1 60	7.766	97.2	77.7
SSEA4	7.988	100.0	79.9
TRA-1-60+SSEA4	7.706	96.5	77.1
Live cell hist	8.513	96.2	85.1

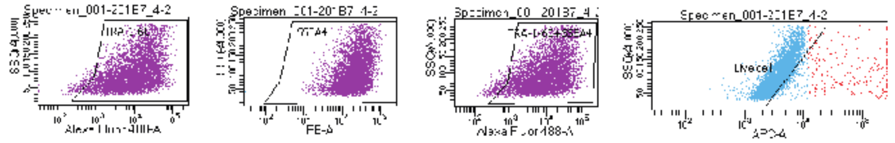

Rep3

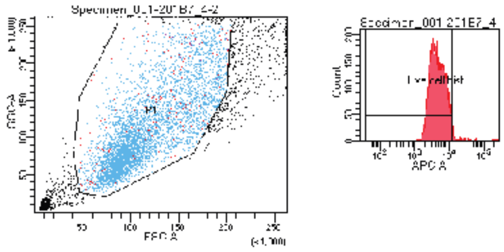

Tube: 201B7 4-2

Population	#Events	%Parent	%Total
All Events	10,000	####	100.0
P1	7,153	71.5	71.5
Live cell	6,208	86.8	62.1
TRA-1-60	5,943	95.7	59.4
SSEA4	6,207	100.0	62.1
TRA-1-60+SSEA4	5,894	95.0	58.9
Live cell hist	6,757	94.5	67.6

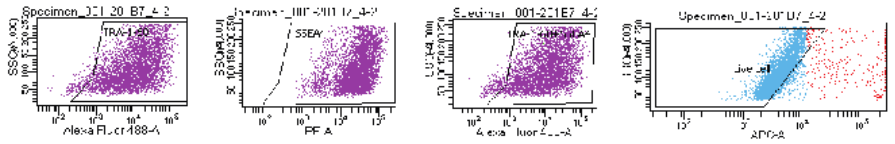

Supplementary Figure 2. Comparison of FCM analysis results with different auto-gating functions.

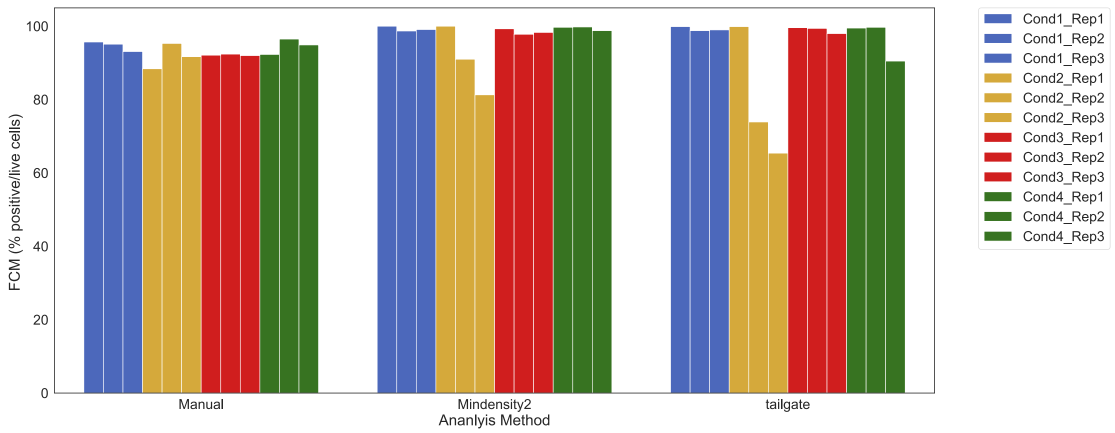

Supplementary Figure 3. Density plots for immunochemistry data. Black dotted lines are the manual thresholds common to all samples for each protein (intensity=100 for NANOG and intensity=80 for OCT4). Red dotted lines are the BASC-based auto thresholds depending on the intensity profile of each sample.

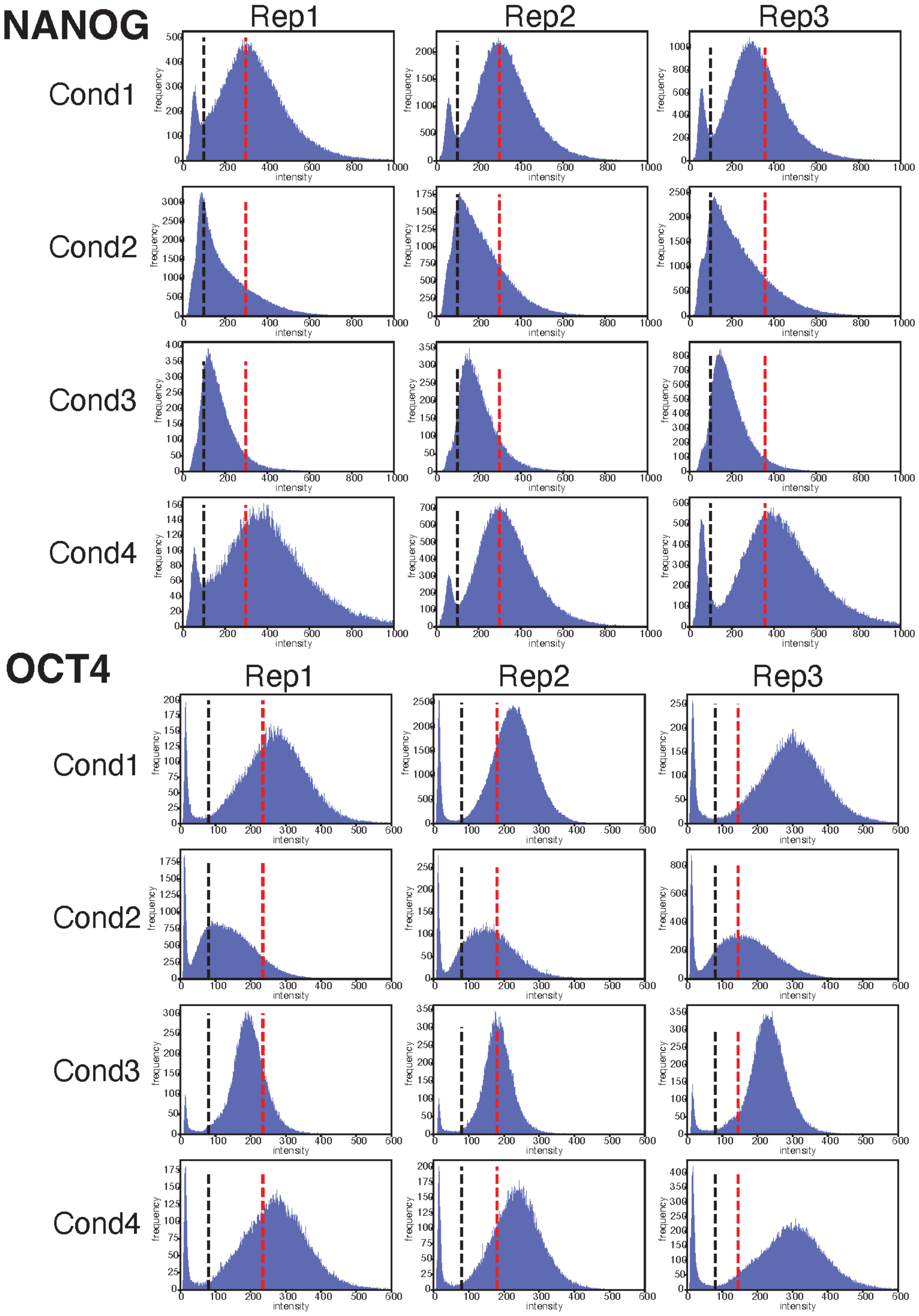

Supplementary Figure 4. Auto gating-based (upper) and Manual-gating (lower) based FCM data analysis for the validation control samples for pluripotency – TRA-1-60 and SSEA-4 double positive cell filtering.

Auto gating

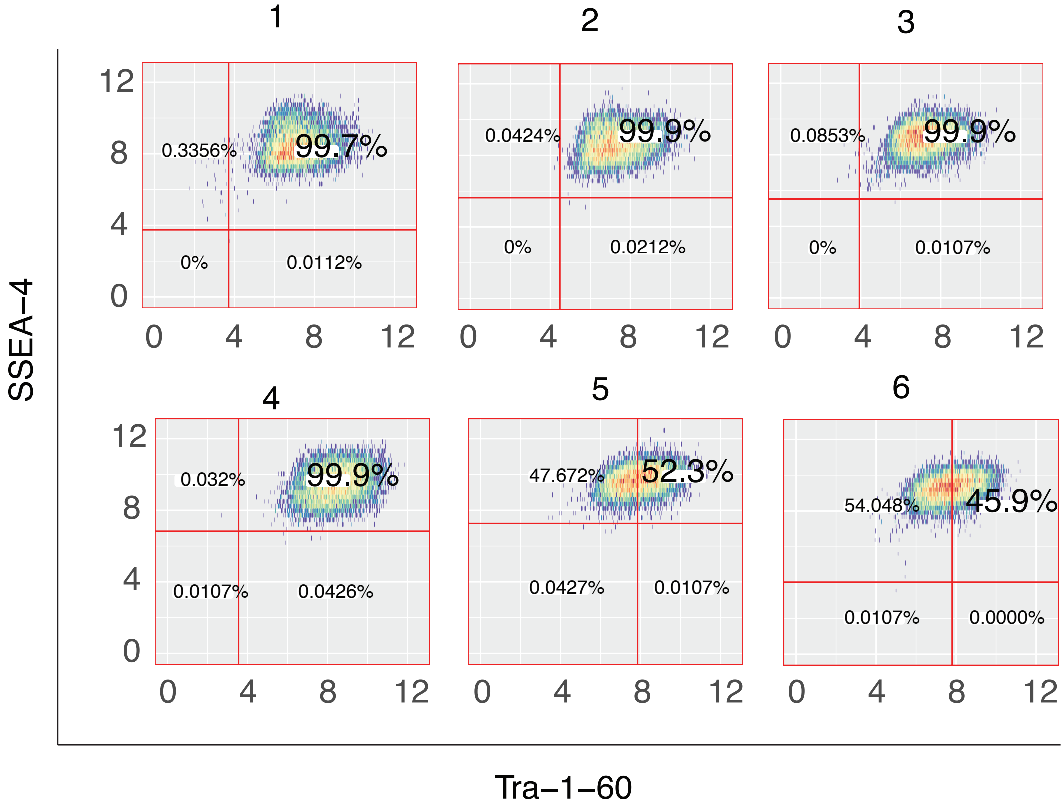

Manual gating

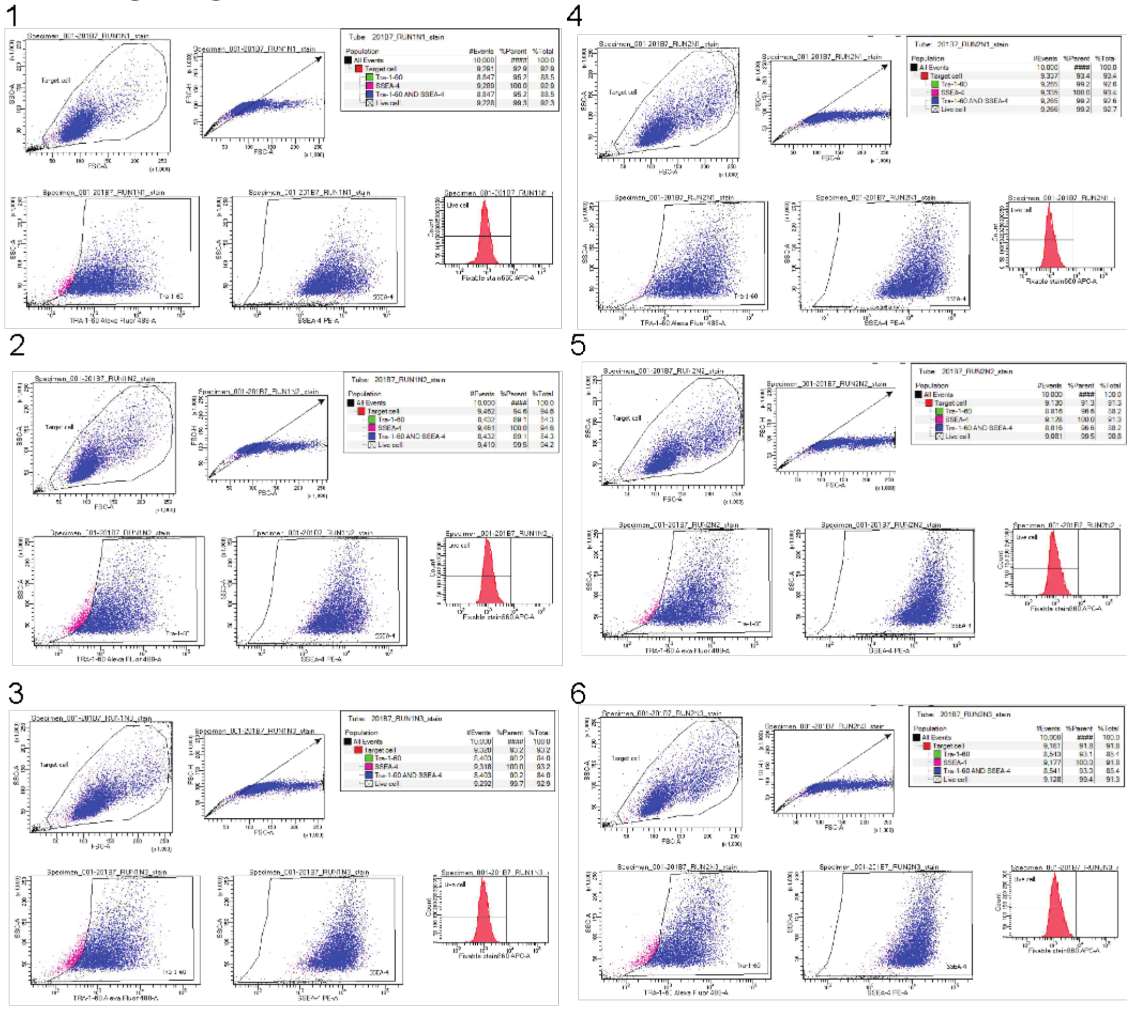

Supplementary Figure 5. Pearson's correlation coefficients between each model prediction and immune-staining results for Nanog or Oct4 positive cells overlayed on UMAP dimension for the model variations based on the predicted pluripotency ratio in 4 conditions, 12 samples.

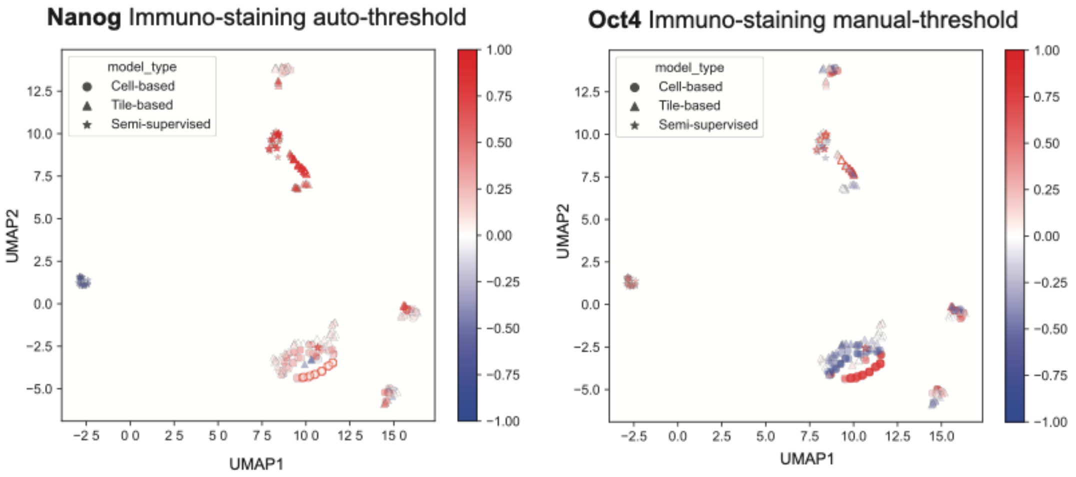

Supplementary Figure 6. Correlation between absolute intensity values in immuno-staining data and the confidence level of model prediction for Nanog (left) and Oct4 (right). Y-axis indicates averaged Oct4 and Nanog intensity for each field of view (FOV) from the immunostaining data for all samples (Cond1-4), and X-axis represents the averaged predicted distance score from the origin of the model (Model III-Tile-based unsupervised model) which uses tiles in the FOV as an input. The higher predicted score means higher confidence of pluripotency and vise-versa.

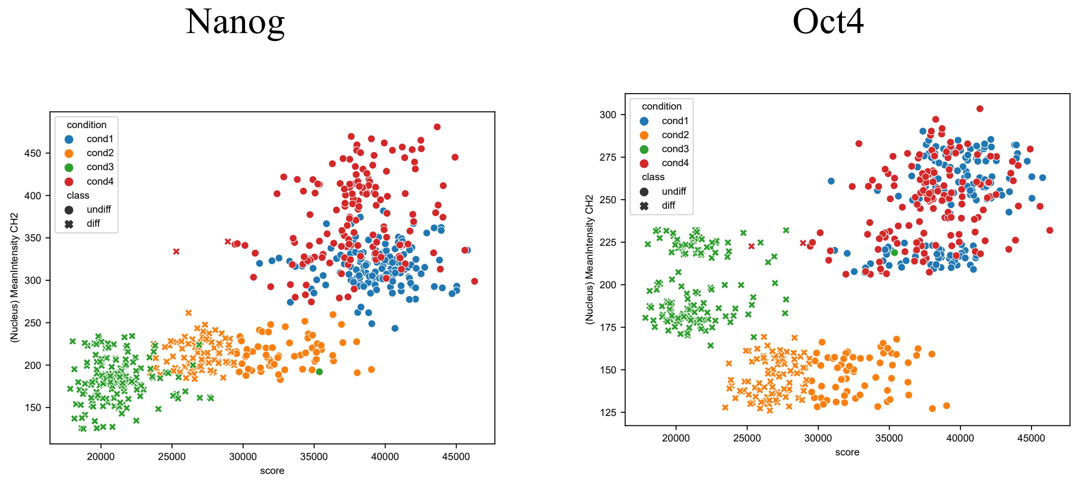

Supplement: Supplementary file 2 — Supplementary Information 2. [file 41598_2024_66591_MOESM2_ESM.pdf]
